# Development of p,q− quasirung orthopair fuzzy hamacher aggregation operators and its application in decision-making problems

**DOI:** 10.1016/j.heliyon.2024.e24726

**Published:** 2024-01-23

**Authors:** Touqeer Ahmad, Muhammad Rahim, Jie Yang, Rabab Alharbi, Hamiden Abd El-Wahed Khalifa

**Affiliations:** a School of Mathematical Sciences, Dalian University of Technology, Dalian 116024, China; b Deparament of Mathematics, Hazara University, Mansehra 21300, PKP, Pakistan; c Department of Mathematics, College of Science and Arts, Qassim University, Ar Rass 51452, Saudi Arabia; d Department of Mathematics, College of Science and Arts, Qassim University, Al-Badaya 51951, Saudi Arabia; e Department of Operations and Management Research, Faculty of Graduate Studies for Statistical Research, Cairo University, Giza 12613, Egypt

**Keywords:** p,q−-quasirung orthopair fuzzy sets, Hamacher t-norm, t-conorm, Aggregation operators, Optimization, Decision making

## Abstract

The concept of p,q− quasirung orthopair fuzzy (p,q− QOF) sets is an advanced extension of q− rung orthopair fuzzy sets (q− ROFSs). This paper introduces the adaptation of Hamacher t-norm and t-conorm to the p,q− QOF environment. A series of Hamacher aggregation operators (AOs) and their associated properties are presented. This study extends its application to multi-criteria group decision-making (MCGDM) for practical problem-solving, illustrated through the analysis of mobile payment platforms. The influence of the aggregation operator parameters, denoted as p and q, on the outcomes of decisions is effectively showcased. Moreover, a comparative analysis is carried out to validate the credibility and authenticity of the proposed model. Finally, the advantages and limitations of the proposed model are outlined.

## 1 Introduction

Multi-criteria decision-making (MCDM) empowers decision-makers to tackle real-world problems more effectively by accommodating complexity, uncertainty, and conflicting objectives. It provides a structured approach to decision-makers that considers the multifaceted nature of modern challenges, resulting in better-informed choices and lasting impact. Fuzzy set (FS) [1] finds wide application in MCDM, adeptly managing the challenges posed by multiple and contradictory criteria. By permitting graded membership for criterion satisfaction, fuzzy MCDM adeptly addressed the uncertainty and subjective nature inherent in decision problems. In a fuzzy set, each element has a membership degree (MD) between 0 and 1, indicating the extent to which the element belongs to the set. Intuitionistic fuzzy sets (IFSs), proposed by Atanassov [2], go further by considering not only the degree of MD but also the non-membership degree (NMD) and the degree of hesitancy for each element. In IFS, for each element *t* the sum of membership (ϑ), and non-membership degree (θ) should not exceed 1, i.e., ϑ+θ≼1. To alleviate the constraints of IFS, Yager [3] introduced Pythagorean fuzzy sets (PFSs) with the stipulation that $\vartheta^{2}+\theta^{2}≼1$. Senapati and Yager [4] introduced Fermatean fuzzy sets (FFSs) and established their core operations, subject to the constraint that $\vartheta^{3}+\theta^{3}≼1$. Yager [5] presented a broader category of such sets referred to as *q* −rung orthopair fuzzy sets (*q* −ROFSs), wherein the sum of the $q^{th}$ power of support and against is bounded by one, i.e., $\vartheta^{q}+\theta^{q}≼1$, for q≽1. q− ROFSs have been effectively introduced and applied across various domains of research, showcasing their versatility and utility in addressing diverse challenges and scenarios. For example, Khan et al. [6] presented an approach to measure data within *q* −ROFSs using the tangent inverse function, coupled with an application in MAGDM utilizing the confidence level approach. Garg et al. [7] presented the notion of dice similarity and generalized dice similarity measures for q-ROFSs, along with the elucidation of fundamental axioms and properties. Farid and Riaz [8] proposed a set of AOs based on Aczel-Alsina operation to aggregate q− ROF information. Kumar and Chen [9] presented q− ROF weighted averaging AO for q− ROF numbers. Jana et al. [10] introduced linguistic q− ROF Choquet integral operators for assessing sustainable urban parcel delivery strategies, demonstrating their qualities and employing them in a MCGDM approach. Mandal et al. [11] introduced a regret theory-based three-way conflict analysis model under q− ROF information, incorporating parameter studies and three-way DM approaches.

Seikh and Mandel [12] devised a series of AOs using Frank t-norm and t-conorm to merge q− ROF information. Garg established [13] a set of AOs referred to as sine trigonometric weighted averaging and geometric operators, intended for aggregating q− ROF information. Further studies regarding q− ROF have been referenced in Refs. [[14], [15], [16], [17], [18]].

Upon examination of the preceding discourse, it is evident that the prevailing theories are delineated within certain confines. For instance, in the context of q− ROF environments, decision-makers face constraints in assigning identical values to q for MD and NMD, a limitation that can exert substantial influence on the overall decision-making process. To address and expand upon these constraints and broaden the applicability, an innovative approach has been introduced. Seikh and Mandal [19] have recently put forth the concept of p,q− quasirung orthopair fuzzy sets (p,q− QOFSs), an extension of *q* –ROF framework. p,q− QOFSs redefine existing paradigms, incorporating two adjustable parameters, namely p and q. These parameters empower decision-makers to tailor MD and NMD in alignment with specific requisites, thereby offering a more adaptable structure to accommodate complex decision scenarios. This evolution holds significant promise in advancing the field by equipping practitioners with a tool to handle intricate decision landscapes, fostering more nuanced and well-informed decision-making processes. Researchers have embraced this structure, introducing diverse approaches based on it. For example, Rahim et al. [20] proposed sine trigonometric operations and their AOs for p,q− QOF numbers (p,q− QOFNs). Ali and Naeem [21]presented Aczel-Alsina AOs in p,q− QOF environment to handle MCDM problems. Chu et al. [22] presented cubic p,q− QOFSs and their basic operational laws. Rahim et al. [23] proposed confidence level-based AOs based on p,q− QOFSs.

### 1.1 Literature review

The Hamacher t-norm and t-conorm were introduced by Hamacher [24] in 1979 as a generalization of the product and sum operations of classical set theory. Hamacher operational laws are parametric operations, meaning they involve a parameter (usually denoted as ***λ***) that controls the level of interaction or aggregation between two values. These properties make them advantageous over traditional operations in a wide range of applications involving uncertain or imprecise information. Hamacher t-norm and t-conorm have garnered significant attention from researchers who have harnessed these operators to devise diverse methodologies tailored for various contextual environments. These operations have been employed as foundational components in a multitude of studies, each contributing unique approaches that leverage the strengths of the Hamacher t-norm and t-conorm to address different challenges and scenarios. For example, Silambarasan and Sriram [25] introduced the operations of Hamacher scalar multiplication and Hamacher exponentiation for Intuitionistic fuzzy matrices. Hadi et al. [26] proposed some operational laws for handling Fermatean fuzzy information by utilizing Hamacher t-norm and t-conorm. They also presented a series of AOs to aggregate Fermatean fuzzy information. Senapati and Chen [27] introduced Hamacher operations for interval-valued PFSs and devised AOs to effectively combine interval-valued Pythagorean fuzzy information. Rawat and Komal [28] formulated a set of Muirhead mean operators for q-ROF numbers by incorporating Hamacher t-norm and t-conorm. Jan et al. [29] presented the notion of Fermatean fuzzy Hamacher prioritized AO and Fermatean fuzzy Hamacher prioritized weighted AO to aggregate the Fermatean fuzzy data. Zedam et al. [30] assessed the conceptual framework of Hamacher's operational laws and their consequential outcomes. Additionally, they delved into the theory of complex T-spherical fuzzy Hamacher weighted averaging and T-spherical fuzzy Hamacher weighted geometric operators, elucidating their significant properties through a series of robust findings. Abdullah et al. [31] established cubic Pythagorean fuzzy AOs through the utilization of Hamacher t-norm and t-conorm, while also outlining their essential properties. Ali et al. [32] introduced power AOs that leverage Hamacher t-norm and t-conorm within the context of complex Intuitionistic fuzzy numbers. Gayen et al. [33] employed Hamacher t-norm and t-conorm to establish core operational laws for q-rung orthopair trapezoidal fuzzy numbers, introducing a series of tailored AOs. Akram et al. [34] introduced the concept of q− ROF Hamacher graphs and Hamacher operators, highlighting their flexibility and parameterization in decision-making. Shahzadi et al. [35] proposed a series of AOs for interval-valued FFS to achieve desired outcomes, which were then applied to address MADM problems.

### 1.2 Motivations

From the earlier discourse, it becomes evident that these operators are formulated within specific constraints. To illustrate, the operators designed for Intuitionistic fuzzy numbers come with the constraint that the sum of MD (ϑ) and NMD (θ) must not surpass 1 i.e., ϑ+θ≼1. Similarly, Pythagorean fuzzy AOs are introduced with the limitation that the square sum of ϑ and θ should be equal to or less than 1 i.e., $\vartheta^{2}+\theta^{2}≼1$. Meanwhile, Fermatean fuzzy AOs are bounded by the condition that the cubic sum of ϑ and θ must not exceed 1 i.e., $\vartheta^{3}+\theta^{3}≼1$. Furthermore, q− rung orthopair fuzzy AOs are stipulated under the prerequisite that the $p^{th}$ power of ϑ and ϑ remains within 1 i.e., $\vartheta^{q}+\theta^{q}≼1$ where q≽1. These constraints possess the potential to exert an influence on the entirety of the decision-making process.

Considering these limitations, we present a suite of aggregation operators designed to mitigate these constraints. Initially, we propose a set of operational laws by Leveraging the Hamacher t-norm and t-conorm. Subsequently, building upon these operations, we introduce a range of aggregation operators, namely p,q− QOF Hamacher weighted averaging (p,q− QOFHWA) and p,q− QOF Hamacher weighted geometric (p,q− QOFHWA) operators. The primary advantage of these operators stems from their parameter-based nature. In the decision-making process, practitioners can effectively manage the influence of membership degree using the parameter p and non-membership with parameter q, tailoring their utilization to suit the specific demands of varying scenarios. Additionally, we delve into an exploration of the impact of the Hamacher parameter λ on decision-making outcomes across different values. This comprehensive analysis serves to enrich our understanding of the nuanced interplay between parameters and outcomes in the decision-making realm.

### 1.3 Objectives

The objectives of the proposed work encompass the following key aspects.

1. To devise a series of novel AOs that effectively address the limitations associated with existing operators in various fuzzy set frameworks, such as IFSs, PFSs, FFSs, and *q* −ROFSs.
2. To introduce AOs that offer enhanced flexibility by relaxing the constraints imposed by the existing operators. These new operators should provide decision-makers with more adaptable tools for handling diverse decision scenarios.
3. To develop AOs that are inherently parametric, allowing decision-makers to fine-tune the influence of membership and non-membership degrees using designed parameters. This parameter-based approach aims to enable a more precise representation of real-world decision contexts.
4. To establish a set of operational laws by employing Hamacher t-norm and t-conorm, which will serve as the foundation for constructing the proposed AOs. These operational laws should contribute to a robust and comprehensive framework for aggregation.
5. To systematically compare and evaluate the performance of the newly proposed AOs against existing methods. This analysis aims to demonstrate the superiority of the proposed operators in terms of their ability to handle limitations, flexibility, and accuracy in decision-making.
6. To conduct an in-depth exploration of the impact of key parameters, such as p, q and λ on decision outcomes within the proposed AOs. This investigation seeks to unveil insights into the interplay between parameters and decision results.
7. To demonstrate the practical utility of the proposed AOs through illustrative examples and case studies in diverse decision-making scenarios. This showcases the effectiveness and versatility of the operators in real-world applications.

**Fig. 1:**
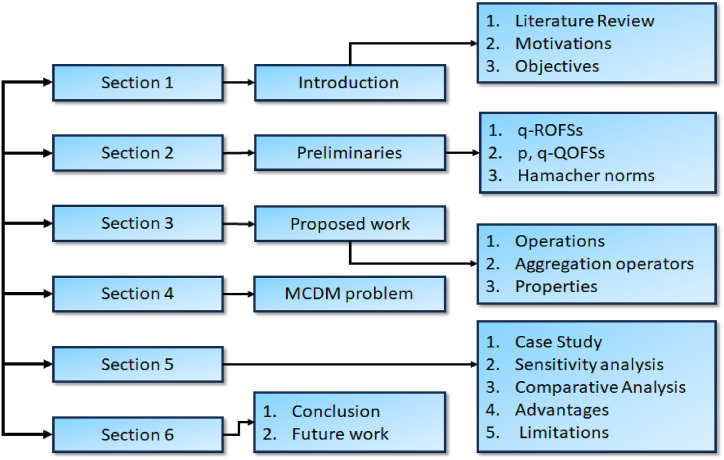
Paper outline.

The structure of the paper is as follows: The succeeding section provides a concise review of fundamental concepts relevant to the presented work. In section 3, we establish novel operational laws using Hamacher norms within p,q− QOFSs and introduce a range of AOs along with their associated properties. In section 4, these operators are harnessed to create practical tools for tackling p,q− QOF multi-criteria group decision-making issues. In section 5, we delve into a case study, specifically the assessment of a cyclone disaster, to illustrate the application of the proposed research. Finally, section 6 presents the conclusion of the proposed work. The comprehensive structure of the paper is delineated in Fig. 1.

## 2 Preliminaries

Within this section, we consolidate the essential understanding pertaining to q− ROFSs, p,q− QOFSs, and Hamacher norms, encompassing their respective operations and relevant properties. We will delve into familiar concepts that hold significance in the subsequent analysis.

### **2.1** **Definition 1.** [5] let D be a non-empty finite set. A q− ROF set Q over an element d∈D can be defined as follows

(1) $$Q=\left\{d,\left\langle \vartheta_{Q}\left(d\right),\theta_{Q}\left(d\right)\right\rangle |d\in D\right\}$$

where $\vartheta_{Q}\left(d\right)\in \left[0,1\right]$ represent MD and $\theta_{Q}\left(d\right)\in \left[0,1\right]$ represent NMD of an element d∈D such that ${\left(\vartheta_{Q}\left(d\right)\right)}^{q}+{\left(\theta_{Q}\left(d\right)\right)}^{q}≼1$ for q≽1. To simplify matters, we denote a q− ROF set presented in Equation (1) as $Q=\left(\vartheta_{Q},\theta_{Q}\right)$, referring to it as q− ROF element or a q− ROF number (q− ROFN). A q-ROFN represents a single element with its MD and NMD, whereas a q-ROFS is a broader collection or set of q-ROFNs.

Definition 2 [5] Let $Q=\left(\vartheta_{Q},\theta_{Q}\right)$ be a q− ROFN. The score function (Sc) of Q can be determined as follows:

(2) $$Sc\left(Q\right)=\vartheta_{Q}^{q}-\theta_{Q}^{q}$$

where −1≼Sc(Q)≼1. In a q− ROFN, a score of −1 indicates strong non-membership, 0 suggests a neutral relationship, and 1 signifies strong membership. Based on the score function, a q− ROFN is favored when it possesses a higher score value. The motivation behind the score function's definition is to quantify the difference between membership and non-membership values, providing a measure of the degree of membership. Let $Q_{1}=\left(0.8,0.5\right)$ and $Q_{2}=\left(0.6,0.3\right)$ are two q− ROFNs. Then for q=2, $Sc\left(Q_{1}\right)=0.39$ and $Sc\left(Q_{2}\right)=0.27$. Thus, $Q_{1}$ is priority over $Q_{2}$. If the score values of two distinct q− ROFNs become identical, it is necessary to compute accuracy values using the accuracy function [5] (Ac), as defined by:

(3) $$Ac\left(Q\right)=\vartheta_{Q}^{q}+\theta_{Q}^{q}$$

where 1≼Ac(Q)≼1 Let $Q_{1}=\left(0.60,0.60\right)$ and $Q_{2}=\left(0.50,0.50\right)$ are two different q− ROFNs. Then by Equation (2) $Sc\left(Q_{1}\right)=0$ and $Sc\left(Q_{2}\right)=0$. Thus, it is necessary to find the accuracy values of $Q_{1}$ and $Q_{2}$ using Equation (3) as follows:

$$Ac\left(Q_{1}\right)=\vartheta_{Q_{1}}^{q}+\theta_{Q_{1}}^{q}=0.60^{2}+0.60^{2}=0.36+0.36=0.72\in \left[0,1\right]\text{.}$$

$$Ac\left(Q_{2}\right)=\vartheta_{Q_{2}}^{q}+\theta_{Q_{2}}^{q}=0.50^{2}+0.50^{2}=0.25+0.25=0.50\in \left[0,1\right]\text{.}$$

Thus, $Q_{1}$ is priority over $Q_{2}$.

Definition 3 [5,36] Let $Q_{1}=\left({\vartheta_{Q}}_{1},{\theta_{Q}}_{2}\right)$, $Q_{1}=\left({\vartheta_{Q}}_{1},{\theta_{Q}}_{2}\right)$ and $Q=\left(\vartheta_{Q},\theta_{Q}\right)$ are any three q− ROFNs. Then their operational laws are defined as follows:

1. $Q^{C}=\left(\theta_{Q},\vartheta_{Q}\right)$;
2. $Q_{1}⋃Q_{2}=\left(\max\left(\vartheta_{Q_{1}},\vartheta_{Q_{2}}\right),\min\left(\theta_{Q_{1}},\theta_{Q_{2}}\right)\right)$;
3. $Q_{1}⋂Q_{2}=\left(\min\left(\vartheta_{Q_{1}},\vartheta_{Q_{2}}\right),\max\left(\theta_{Q_{1}},\theta_{Q_{2}}\right)\right)$;
4. $Q_{1}⊕Q_{2}=\left(\sqrt[{q}]{\vartheta_{Q_{1}}^{q}+\vartheta_{Q_{2}}^{q}-\vartheta_{Q_{1}}^{q}\vartheta_{Q_{2}}^{q}},\theta_{Q_{1}}\theta_{Q_{2}}\right)$;
5. $Q_{1}⊕Q_{2}=\left(\vartheta_{Q_{1}}\vartheta_{Q_{2}},\sqrt[{q}]{\theta_{Q_{1}}^{q}+\theta_{Q_{2}}^{q}-\theta_{Q_{1}}^{q}\theta_{Q_{2}}^{q}}\right)$;
6. $Q^{\eta }=\left(\vartheta_{Q}^{\eta },\sqrt[{q}]{1-{\left(1-\theta_{Q}^{q}\right)}^{\eta }}\right)$;
7. $\eta Q=\left(\sqrt[{q}]{1-{\left(1-\vartheta_{Q}^{q}\right)}^{\eta }},\theta_{Q}^{\eta }\right)$; where η≻1.

### **2.2** p,q−**QOF sets**

Definition 4 [19] Let D be a non-empty finite set. A p,q− QOF set I over an element d∈D can be defined as follows:

(4) $$I=\left\{d,\left\langle \vartheta_{I}\left(d\right),\theta_{I}\left(d\right)\right\rangle |d\in D\right\}$$

where $\vartheta_{I}\left(d\right)\in \left[0,1\right]$ represent MD and $\theta_{I}\left(d\right)\in \left[0,1\right]$ represent NMD of an element d∈D such that ${\left(\vartheta_{I}\left(d\right)\right)}^{p}+{\left(\theta_{I}\left(d\right)\right)}^{q}≼1$ for p, q≽1. To simplify matters, we denote a p,q− QOF set defined in Equation (4) as $Q=\left(\vartheta_{I},\theta_{I}\right)$, referring to it as p,q− QOF element or a p,q− QOF number (p,q− QOFN).

Remark 1 [19] In Definition 4, the value of p can be either greater than, equal to, or less than q. This implies that the value of p and q can be adapted based on the specific circumstances.

Definition 5 [19] Let $I_{1}=\left({\vartheta_{I}}_{1},{\theta_{I}}_{2}\right)$, $I_{1}=\left({\vartheta_{I}}_{1},{\theta_{I}}_{2}\right)$ and $I=\left(\vartheta_{I},\theta_{I}\right)$ are any three q− ROFNs. Then their operational laws for η≻0 are defined as follows:

1. $I^{C}=\left(\theta_{I},\vartheta_{I}\right)$;
2. $I_{1}⋃I_{2}=\left(\max\left(\vartheta_{I_{1}},\vartheta_{I_{2}}\right),\min\left(\theta_{I_{1}},\theta_{I_{2}}\right)\right)$;
3. $I_{1}⋂I_{2}=\left(\min\left(\vartheta_{I_{1}},\vartheta_{I_{2}}\right),\max\left(\theta_{I_{1}},\theta_{I_{2}}\right)\right)$;
4. $I_{1}⊕I_{2}=\left(\sqrt[{p}]{\vartheta_{I_{1}}^{p}+\vartheta_{I_{2}}^{p}-\vartheta_{I_{1}}^{p}\vartheta_{I_{2}}^{p}},\theta_{I_{1}}\theta_{I_{2}}\right)$;
5. $I_{1}⊗I_{2}=\left(\vartheta_{I_{1}}\vartheta_{I_{2}},\sqrt[{q}]{\theta_{I_{1}}^{q}+\theta_{I_{2}}^{q}-\theta_{I_{1}}^{q}\theta_{I_{2}}^{q}}\right)$;
6. $I^{\eta }=\left(\vartheta_{I}^{\eta },\sqrt[{q}]{1-{\left(1-\theta_{I}^{q}\right)}^{\eta }}\right)$;
7. $\eta I=\left(\sqrt[{p}]{1-{\left(1-\vartheta_{I}^{p}\right)}^{\eta }},\theta_{I}^{\eta }\right)$.

Example 1 Let $I_{1}=\left(0.7,0.8\right)$ and $I_{2}=\left(0.9,0.3\right)$ are any two p,q− QOFNs. If p=q=4 and η=0.6, then the operational laws defined in Definition 5 can be calculated as:

$$I_{1}⊕I_{2}=\left(\sqrt[{4}]{{\left(0.7\right)}^{4}+{\left(0.9\right)}^{4}-{\left(0.7\right)}^{4}{\left(0.9\right)}^{4}},\left(0.8\right)\times \left(0.3\right)\right)=\left(0.9271,0.2400\right)\text{,}$$

$$I_{1}⊗I_{2}=\left(\left(0.7\right)\times \left(0.9\right)\sqrt[{4}]{{\left(0.8\right)}^{4}+{\left(0.3\right)}^{4}-{\left(0.8\right)}^{4}{\left(0.3\right)}^{4}}\right)=\left(0.6300,0.8023\right)\text{,}$$

$$I_{1}^{\eta }=\left({\left(0.7\right)}^{0.6},\sqrt[{4}]{1-{\left(1-{\left(0.8\right)}^{4}\right)}^{0.6}}\right)=\left(0.8073,0.7216\right)\text{,}$$

$$\eta I=\left(\sqrt[{4}]{1-{\left(1-{\left(0.7\right)}^{4}\right)}^{0.6}},{\left(0.8\right)}^{0.6}\right)=\left(0.6243,0.8747\right)\text{.}$$

Definition 6 [19] Let $J=\left(\vartheta_{I},\theta_{I}\right)$ be a p,q− QOFN. The score function (Sc) of I can be determined as follows:

(5) $$Sc\left(I\right)=\frac{1+\theta_{I}^{p}-\theta_{I}^{q}}{2}$$

where 0≼Sc(I)≼1. Let $I_{1}=\left(0.6,0.3\right)$ and $I_{1}=\left(0.5,0.4\right)$ are two p,q− QOFNs. For p=q=2, the score values of $I_{1}$ and $I_{2}$ can be determined as follows:

$$Sc\left(I_{1}\right)=\frac{1+\theta_{I}^{p}-\theta_{I}^{q}}{2}=\frac{1+{\left(0.6\right)}^{2}-{\left(0.3\right)}^{2}}{2}=0.6350\text{,}$$

$$Sc\left(I_{2}\right)=\frac{1+\theta_{I}^{p}-\theta_{I}^{q}}{2}=\frac{1+{\left(0.5\right)}^{2}-{\left(0.4\right)}^{2}}{2}=0.5450\text{.}$$

Thus, $I_{1}$ is priority over $I_{2}$.

If the score values of two distinct p,q− QOFNs become identical, it is necessary to compute accuracy values using the accuracy values using the accuracy function [19] (Ac) defined as:

(6) $$Ac\left(J\right)=\theta_{I}^{p}+\theta_{I}^{q}$$

where 1≼Ac(J)≼1, and p and q are any positive integers.

Let $I_{1}=\left(0.40,0.40\right)$ and $I_{1}=\left(0.50,0.50\right)$ are two p,q− QOFNs. For p=q=2, the score values of $I_{1}$ and $I_{2}$ can be determined as follows:

$$Sc\left(I_{1}\right)=\frac{1+\theta_{I}^{p}-\theta_{I}^{q}}{2}=\frac{1+{\left(0.6\right)}^{2}-{\left(0.3\right)}^{2}}{2}=0.5000,Sc\left(I_{2}\right)=\frac{1+\theta_{I}^{p}-\theta_{I}^{q}}{2}=\frac{1+{\left(0.6\right)}^{2}-{\left(0.3\right)}^{2}}{2}=0.5000\text{.}$$

Then by Equation (5) $Sc\left(I_{1}\right)=0.50$ and $Sc\left(I_{2}\right)=0.50$. Thus, it is necessary to find the accuracy values of $I_{1}$ and $I_{2}$ for the comparison of these two p,q− QOFNs by using Equation (6) as follows:

$$Ac\left(I_{1}\right)=\frac{1+\theta_{I}^{p}+\theta_{I}^{q}}{2}=\frac{1+{\left(0.40\right)}^{2}+{\left(0.40\right)}^{2}}{2}=0.6600,Ac\left(I_{2}\right)=\frac{1+\theta_{I}^{p}+\theta_{I}^{q}}{2}=\frac{1+{\left(0.50\right)}^{2}+{\left(0.50\right)}^{2}}{2}=0.7500\text{.}$$

Thus, $I_{2}$ is priority over $I_{1}$.

Definition 7 [19] Let $I_{1}=\left(\vartheta_{I_{1}},\theta_{I_{1}}\right)$, and $I_{2}=\left(\vartheta_{I_{2}},\theta_{I_{2}}\right)$ be any two p,q− QOFNs, then

i. If $Sc\left(I_{1}\right)≻Sc\left(I_{2}\right)$, then $I_{1}≻I_{2}$,
ii. If $Sc\left(I_{1}\right)≺Sc\left(I_{2}\right)$, then $I_{1}≺I_{2}$,
iii. If $Sc\left(I_{1}\right)=Sc\left(I_{2}\right)$, then
  • If $Ac\left(I_{1}\right)≻Ac\left(I_{2}\right)$, then $I_{1}≻I_{2}$,
  • If $Ac\left(I_{1}\right)≻Ac\left(I_{2}\right)$, then $I_{1}≻I_{2}$,
  • If $Ac\left(I_{1}\right)=Ac\left(I_{2}\right)$, then $I_{1}∼I_{2}$.

Definition 8 [24] Suppose $\varphi_{1}$, $\varphi_{2}$, $\psi_{1}$ and $\psi_{2}$ as elements from real numbers. Subsequently, the expression for Hamacher t-norm (T) and t-conorm $\left(T^{*}\right)$ can be expressed as follows:

(7) $$T\left(\varphi_{1},\psi_{1}\right)=\varphi_{1}⨂\varphi_{1}=\frac{\varphi_{1}\psi_{1}}{\lambda +\left(1-\lambda \right)\left(\varphi_{1}+\psi_{1}-\varphi_{1}\psi_{1}\right)},\lambda ≻0$$

(8) $$T^{*}\left(\varphi_{2},\psi_{2}\right)=\varphi_{2}⨁\psi_{2}=\frac{\varphi_{2}+\psi_{2}-\varphi_{2}\psi_{2}-\left(1-\lambda \right)\varphi_{2}\psi_{2}}{1-\left(1-\lambda \right)\varphi_{2}\psi_{2}},\lambda ≻0$$

## 3 Proposed operational laws and AOs

Within this section, we establish a set of operational laws by employing Equations (7), (8) within the context of the p,q− QOF environment. Building upon these defined operational laws, we introduce a comprehensive array of AOs designed specifically for aggregating p,q− QOF information. This approach enhances the adaptability and precision of aggregation processes within this specific framework, thereby contributing to more effective decision-making in complex scenarios.

### 3.1 Operational laws

Definition 9 Let $I_{1}=\left(\vartheta_{I_{1}},\vartheta_{I_{1}}\right)$ and $I_{2}=\left(\vartheta_{I_{2}},\theta_{I_{2}}\right)\text{,}$ be any two p,q− QOFNs, where λ≻0 and η≻0, the essential Hamacher operations for p,q− QOFNs are presented as follows:

i. $I_{1}⨁I_{2}=\left(\sqrt[{p}]{\frac{\vartheta_{I_{1}}^{p}+\vartheta_{I_{2}}^{p}-\vartheta_{I_{1}}^{p}\vartheta_{I_{2}}^{p}-\left(1-\lambda \right)\vartheta_{I_{1}}^{p}\vartheta_{I_{2}}^{p}}{1-\left(1-\lambda \right)\vartheta_{I_{1}}^{p}\vartheta_{I_{2}}^{p}}},\frac{\theta_{I_{1}}\;\theta_{I_{2}}}{\sqrt[{q}]{1-\left(1-\lambda \right)\theta_{I_{1}}^{q}+\theta_{I_{2}}^{q}-\theta_{I_{1}}^{q}\theta_{I_{2}}^{q}\;}}\right)$;
ii. $I_{1}⨂I_{2}=\left(\frac{\vartheta_{I_{1}}\;\vartheta_{I_{2}}}{\sqrt[{p}]{1-\left(1-\lambda \right)\vartheta_{I_{1}}^{p}+\vartheta_{I_{2}}^{p}-\vartheta_{I_{1}}^{p}\vartheta_{I_{2}}^{p}\;}},\sqrt[{q}]{\frac{\theta_{I_{1}}^{q}+\theta_{I_{2}}^{q}-\theta_{I_{1}}^{q}\theta_{I_{2}}^{q}-\left(1-\lambda \right)\theta_{I_{1}}^{q}\theta_{I_{2}}^{q}}{1-\left(1-\lambda \right)\theta_{I_{1}}^{q}\theta_{I_{2}}^{q}}}\right)$;
iii. $\eta I_{1}=\left(\;\sqrt[{p}]{\frac{{\left(1+\left(1-\lambda \right)-\left(1-\vartheta_{I_{1}}^{p}\right)\right)}^{\eta }-{\left(\left(1-\vartheta_{I_{1}}^{p}\right)\right)}^{\eta }}{{\left(1+\left(1-\lambda \right)-\left(1-\vartheta_{I_{1}}^{p}\right)\right)}^{\eta }+\left(1-\lambda \right){\left(1-\vartheta_{I_{1}}^{p}\right)}^{\eta }}},\frac{\sqrt[{q}]{\lambda }\theta_{I_{1}}^{\eta }}{\sqrt[{q}]{{\left(1+\left(\lambda -1\right)\left(1-\theta_{I_{1}}^{q}\right)\right)}^{\eta }+\left(\lambda -1\right){\left(\theta_{I_{1}}^{q}\right)}^{\eta }}}\right)$;
iv. $I_{1}^{\eta }=\left(\;\frac{\sqrt[{p}]{\lambda }\vartheta_{I_{1}}^{\eta }}{\sqrt[{p}]{{\left(1+\left(\lambda -1\right)\left(1-\vartheta_{I_{1}}^{p}\right)\right)}^{\eta }+\left(\lambda -1\right){\left(\vartheta_{I_{1}}^{p}\right)}^{\eta }}},\sqrt[{q}]{\frac{{\left(1+\left(1-\lambda \right)-\left(1-\theta_{I_{1}}^{q}\right)\right)}^{\eta }-{\left(\left(1-\theta_{I_{1}}^{q}\right)\right)}^{\eta }}{{\left(1+\left(1-\lambda \right)-\left(1-\theta_{I_{1}}^{q}\right)\right)}^{\eta }+\left(1-\lambda \right){\left(1-\theta_{I_{1}}^{q}\right)}^{\eta }}}\right)$.

Example 2 Let $I_{1}=\left(0.5,0.4\right)$ and $I_{2}=(0.8,0.3$^)^ are two p,q− QOFNs. If p=q=4, η=0.6 and λ=1, then the operational laws defined Definition 9 can be calculated as:

$$I_{1}⨁I_{2}=\left(\sqrt[{p}]{\frac{\vartheta_{I_{1}}^{p}+\vartheta_{I_{2}}^{p}-\vartheta_{I_{1}}^{p}\vartheta_{I_{2}}^{p}-\left(1-\lambda \right)\vartheta_{I_{1}}^{p}\vartheta_{I_{2}}^{p}}{1-\left(1-\lambda \right)\vartheta_{I_{1}}^{p}\vartheta_{I_{2}}^{p}}},\frac{\theta_{I_{1}}\;\theta_{I_{2}}}{\sqrt[{q}]{1-\left(1-\lambda \right)\theta_{I_{1}}^{q}+\theta_{I_{2}}^{q}-\theta_{I_{1}}^{q}\theta_{I_{2}}^{q}\;}}\right)$$

$$=\left(\sqrt[{4}]{\frac{0.5^{4}+0.8^{4}-\;0.5^{4}0.8^{4}-\left(1-1\right)0.5^{4}0.8^{4}}{1-\left(1-1\right)0.5^{4}0.8^{4}}},\frac{\left(0.4\right)\left(0.3\right)}{\sqrt[{4}]{1-\left(1-1\right)0.4^{4}+0.3^{4}-0.4^{4}0.3^{4}\;}}\right),=\left(0.8174,0.1200\right)\text{.}$$

$$I_{1}⨂I_{2}=\left(\frac{\vartheta_{I_{1}}\;\vartheta_{I_{2}}}{\sqrt[{p}]{1-\left(1-\lambda \right)\vartheta_{I_{1}}^{p}+\vartheta_{I_{2}}^{p}-\vartheta_{I_{1}}^{p}\vartheta_{I_{2}}^{p}\;}},\sqrt[{q}]{\frac{\theta_{I_{1}}^{q}+\theta_{I_{2}}^{q}-\theta_{I_{1}}^{q}\theta_{I_{2}}^{q}-\left(1-\lambda \right)\theta_{I_{1}}^{q}\theta_{I_{2}}^{q}}{1-\left(1-\lambda \right)\theta_{I_{1}}^{q}\theta_{I_{2}}^{q}}}\right)$$

$$=\left(\frac{0.5^{4}0.8^{4}}{\sqrt[{4}]{1-\left(1-1\right)0.5^{4}+0.8^{4}-0.5^{4}0.8^{4}\;}},\sqrt[{4}]{\frac{0.4^{4}+0.3^{4}-0.4^{4}0.3^{4}-\left(1-\lambda \right)0.4^{4}0.3^{4}}{1-\left(1-1\right)0.4^{4}0.3^{4}}}\right)={\left(0.4000,0.4278\right)}_{,}$$

$$\eta I_{1}=\left(\;\sqrt[{p}]{\frac{{\left(1+\left(1-\lambda \right)-\left(1-\vartheta_{I_{1}}^{p}\right)\right)}^{\eta }-{\left(\left(1-\vartheta_{I_{1}}^{p}\right)\right)}^{\eta }}{{\left(1+\left(1-\lambda \right)-\left(1-\vartheta_{I_{1}}^{p}\right)\right)}^{\eta }+\left(1-\lambda \right){\left(1-\vartheta_{I_{1}}^{p}\right)}^{\eta }}},\frac{\sqrt[{q}]{\lambda }\theta_{I_{1}}^{\eta }}{\sqrt[{q}]{{\left(1+\left(\lambda -1\right)\left(1-\theta_{I_{1}}^{q}\right)\right)}^{\eta }+\left(\lambda -1\right){\left(\theta_{I_{1}}^{q}\right)}^{\eta }}}\right)$$

$$=\left(\begin{array}{c} \sqrt[{4}]{\frac{{\left(1+\left(1-1\right)-\left(1-0.5^{4}\right)\right)}^{0.6}-{\left(\left(1-0.5^{4}\right)\right)}^{0.6}}{{\left(1+\left(1-1\right)-\left(1-0.5^{4}\right)\right)}^{0.6}+\left(1-1\right){\left(1-0.5^{4}\right)}^{0.6}}},\\ \frac{\sqrt[{4}]{1}{\left(0.4\right)}^{0.6}}{\sqrt[{q}]{{\left(1+\left(1-1\right)\left(1-0.4^{4}\right)\right)}^{0.6}+\left(1-1\right){\left(0.4^{4}\right)}^{0.6}}} \end{array}\right)=\left(0.4415,0.5771\right)\text{.}$$

$$I_{1}^{\eta }=\left(\;\frac{\sqrt[{p}]{\lambda }\vartheta_{I_{1}}^{\eta }}{\sqrt[{p}]{{\left(1+\left(\lambda -1\right)\left(1-\vartheta_{I_{1}}^{p}\right)\right)}^{\eta }+\left(\lambda -1\right){\left(\vartheta_{I_{1}}^{p}\right)}^{\eta }}},\sqrt[{q}]{\frac{{\left(1+\left(1-\lambda \right)-\left(1-\theta_{I_{1}}^{q}\right)\right)}^{\eta }-{\left(\left(1-\theta_{I_{1}}^{q}\right)\right)}^{\eta }}{{\left(1+\left(1-\lambda \right)-\left(1-\theta_{I_{1}}^{q}\right)\right)}^{\eta }+\left(1-\lambda \right){\left(1-\theta_{I_{1}}^{q}\right)}^{\eta }}}\right)\text{;}$$

$$=\left(\begin{array}{c} \frac{\sqrt[{4}]{1}{\left(0.5\right)}^{0.6}}{\sqrt[{4}]{{\left(1+\left(\lambda -1\right)\left(1-0.5^{4}\right)\right)}^{0.6}+\left(\lambda -1\right){\left(0.5\right)}^{0.6}}},\\ \sqrt[{4}]{\frac{{\left(1+\left(1-1\right)-\left(1-0.4\right)\right)}^{0.6}-{\left(\left(1-0.4\right)\right)}^{0.6}}{{\left(1+\left(1-1\right)-\left(1-0.4\right)\right)}^{0.6}+\left(1-1\right){\left(1-0.4\right)}^{0.6}}} \end{array}\right)=\left(0.6598,0.3525\right)\text{.}$$

### 3.2 p,q –QOFHWA operators

Definition 10 Assuming $I_{i}=\left(\vartheta_{I_{i}},\theta_{I_{i}}\right)$ (i=1,2,…,n) be a collection of p,q –QOFNs, the p,q –QOF Hamacher weighted averaging (p,q –QOFHWA) operator is defined as a mapping p,q –QOFHWA: $I^{n}\to I\text{.}$ Characterized by:

$$p,q\;–\text{QOFHWA}\;\left(I_{1},I_{2},\ldots ,I_{n}\right)={⨁}_{i=1}^{n}\left(\psi_{i}I_{i}\right)$$

(9) $$=\left(\frac{\sqrt[{p}]{\frac{\prod_{i=1}^{n}{\left(1+\left(\lambda -1\right)\vartheta_{I_{i}}^{p}\right)}^{w_{i}}-\prod_{i=1}^{n}{\left(1-\vartheta_{I_{i}}^{p}\right)}^{w_{i}}}{\prod_{i=1}^{n}{\left(1+\left(1-\lambda \right)\vartheta_{I_{i}}^{p}\right)}^{w_{i}}+\left(\lambda -1\right)\prod_{i=1}^{n}{\left(1-\vartheta_{I_{i}}^{p}\right)}^{w_{i}}},\sqrt[{q}]{\lambda }\prod_{i=1}^{n}\vartheta_{I_{i}}^{w_{i}}}}{\sqrt[{q}]{\prod_{i=1}^{n}{(1+\left(\lambda -1\right)(1-\theta_{I_{i}}^{q}))}^{w_{i}}+}\left(\lambda -1\right)\prod_{i=1}^{n}{\left(\theta_{I_{i}}^{q}\right)}^{w_{i}}}\right)$$

Here, $w={\left(w_{1},w_{2},\ldots ,w_{n}\right)}^{T}$ signifies the weight vector of $I_{i}=\left(\vartheta_{I_{i}},\theta_{I_{i}}\right)$ (i=1,2,…,n) adhering the conditions $w_{i}≻0$ and the constrain $\sum_{i=1}^{n}w_{i}=1$.

Theorem 1 Assuming $I_{i}=\left(\vartheta_{I_{i}},\theta_{I_{i}}\right)$ (i=1,2,…,n) be a collection of p,q –QOFNs, it follows that the aggregation values of this family achieved through the p,q –QOFHWA operator, is also a p,q –*QOFNs*.

***Proof*.** This proof can be readily demonstrated through mathematical induction based on the variable *n*.

***Step* 1.** When n=1, the value of $w_{1}$ become 1, and by examining the left side Equation (9), we obtain:

p,q –QOFHWA $\left(I_{1},I_{2},\ldots ,I_{n}\right)=I_{1}=\left(\vartheta_{I_{1}},\theta_{I_{1}}\right)$, similarly, for the right-hand side of Equation (9), we get

$$\left(\sqrt[{p}]{\frac{\left(1+\left(\lambda -1\right)\vartheta_{I_{1}}^{p}\right)-\left(1-\vartheta_{I_{1}}^{p}\right)}{1+\left(1-\lambda \right)\vartheta_{I_{1}}^{p}+\left(\lambda -1\right)\left(1-\theta_{I_{1}}^{p}\right)},\frac{\sqrt[{q}]{\lambda \theta_{I_{1}}}}{\sqrt[{q}]{1+\left(\lambda -1\right)\left(1-\theta_{I_{1}}^{q}\right)+}\left(\lambda -1\right)\theta_{I_{1}}^{q}}}\right)=\left(\vartheta_{I_{1}},\theta_{I_{1}}\right)\text{.}$$

Therefore, Equation (9) is valid when n=1.

***Step* 2.** Suppose that Equation (9) holds for the case of n=k, where k is any real number. In this scenario, Equation (8) can be expressed as:

$$p,q\;–\text{QOFHWA}=\left(\begin{array}{c} \sqrt[{p}]{\frac{\prod_{i=1}^{k}{\left(1+\left(\lambda -1\right)\vartheta_{I_{i}}^{p}\right)}^{w_{i}}-\prod_{i=1}^{k}{\left(1-\vartheta_{I_{i}}^{p}\right)}^{w_{i}}}{\prod_{i=1}^{n}{\left(1+\left(1-\lambda \right)\vartheta_{I_{i}}^{p}\right)}^{w_{i}}+\left(\lambda -1\right)\prod_{i=1}^{n}{\left(1-\vartheta_{I_{i}}^{p}\right)}^{w_{i}}},}\\ \frac{\sqrt[{q}]{\lambda }\prod_{i=1}^{k}\theta_{I_{i}}^{w_{i}}}{\sqrt[{q}]{\prod_{i=1}^{k}{(1+\left(\lambda -1\right)(1-\theta_{I_{i}}^{q}))}^{w_{i}}+}\left(\lambda -1\right)\prod_{i=1}^{k}{\left(\theta_{I_{i}}^{q}\right)}^{w_{i}}} \end{array}\right)$$

***Step* 3.** Now, when considering the situation where k=n+1, we examine the following Equations.

$$p,q\;–\text{QOFHWA}\;\left(I_{1},I_{2},\ldots ,I_{k}\right)={⨁}_{i=1}^{k}\left(\psi_{i}I_{i}\right)⨁\left(\psi_{k+1}I_{k+1}\right)$$

$$=\left(\begin{array}{c} \sqrt[{p}]{\frac{\prod_{i=1}^{k}{\left(1+\left(\lambda -1\right)\vartheta_{I_{i}}^{p}\right)}^{w_{i}}-\prod_{i=1}^{k}{\left(1-\vartheta_{I_{i}}^{p}\right)}^{w_{i}}}{\prod_{i=1}^{n}{\left(1+\left(1-\lambda \right)\vartheta_{I_{i}}^{p}\right)}^{w_{i}}+\left(\lambda -1\right)\prod_{i=1}^{n}{\left(1-\vartheta_{I_{i}}^{p}\right)}^{w_{i}}},}\\ \frac{\sqrt[{q}]{\lambda }\prod_{i=1}^{k}\theta_{I_{i}}^{w_{i}}}{\sqrt[{q}]{\prod_{i=1}^{k}{\left(1+\left(\lambda -1\right)\left(1-\theta_{I_{i}}^{q}\right)\right)}^{w_{i}}+}\left(\lambda -1\right)\prod_{i=1}^{k}{\left(\theta_{I_{i}}^{q}\right)}^{w_{i}}} \end{array}\right)$$

$$⨁\left(\frac{\sqrt[{p}]{\frac{{\left(1+\left(\lambda -1\right)\vartheta_{I_{k+1}}^{p}\right)}^{w_{k+1}}-{\left(1-\vartheta_{I_{k+1}}^{p}\right)}^{w_{k+1}}}{{\left(1+\left(1-\lambda \right)\vartheta_{I_{k+1}}^{p}\right)}^{w_{k+1}}+\left(\lambda -1\right){\left(1-\vartheta_{I_{k+1}}^{p}\right)}^{w_{k+1}}},\sqrt[{q}]{\lambda }\theta_{I_{k+1}}^{w_{k+1}}}}{\sqrt[{q}]{{\left(1+\left(\lambda -1\right)\left(1-\theta_{I_{k+1}}^{q}\right)\right)}^{w_{k+1}}+}\left(\lambda -1\right){\left(\theta_{I_{k+1}}^{q}\right)}^{w_{k+1}}}\;\right)$$

$$=\left(\begin{array}{c} \sqrt[{p}]{\frac{\prod_{i=1}^{k+1}{\left(1+\left(\lambda -1\right)\vartheta_{I_{i}}^{p}\right)}^{w_{i}}-\prod_{i=1}^{k+1}{\left(1-\vartheta_{I_{i}}^{p}\right)}^{w_{i}}}{\prod_{i=1}^{n}{\left(1+\left(1-\lambda \right)\vartheta_{I_{i}}^{p}\right)}^{w_{i}}+\left(\lambda -1\right)\prod_{i=1}^{n}{\left(1-\vartheta_{I_{i}}^{p}\right)}^{w_{i}}},}\\ \frac{\sqrt[{q}]{\lambda }\prod_{i=1}^{k+1}\theta_{I_{i}}^{w_{i}}}{\sqrt[{q}]{\prod_{i=1}^{k+1}{\left(1+\left(\lambda -1\right)\left(1-\theta_{I_{i}}^{q}\right)\right)}^{w_{i}}+}\left(\lambda -1\right)\prod_{i=1}^{k+1}{\left(\theta_{I_{i}}^{q}\right)}^{w_{i}}} \end{array}\right)$$

Thus, it can be concluded that Equation (9) remains valid for the case of k=n+1. By combining the insight from step (1), (2), and (3), it becomes evident that this outcome extends to any values of n within the set of natural numbers.

Example 3 Consider the four p,q –QOFNs: $I_{1}=\left(0.5,0.4\right).\;I_{2}=\left(0.5,0.3\right),I_{3}=\left(0.6,0.7\right)\;and\;I_{4}=\left(0.4,0.6\right)\text{.}$ Let $w={\left(0.3,0.1,0.4,0.2\right)}^{T}$ represent the weight vector for these p,q –QOFNs. We assume λ=2 and p=q=2, then

$$\prod_{i=1}^{n}{\left(1+\left(\lambda -1\right)\vartheta_{I_{i}}^{p}\right)}^{w_{i}}={\left(1+\left(2-1\right){\left(0.5\right)}^{2}\right)}^{0.3}{\left(1+\left(2-1\right){\left(0.5\right)}^{2}\right)}^{0.1}{\left(1+\left(2-1\right){\left(0.6\right)}^{2}\right)}^{0.4}{\left(1+\left(2-1\right){\left(0.4\right)}^{2}\right)}^{0.2}=1.2929\text{.}$$

$$\prod_{i=1}^{n}{\left(1-\vartheta_{I_{i}}^{p}\right)}^{w_{i}}={\left(1+{\left(0.5\right)}^{2}\right)}^{0.3}{\left(1+{\left(0.5\right)}^{2}\right)}^{0.1}{\left(1+{\left(0.6\right)}^{2}\right)}^{0.4}{\left(1+{\left(0.4\right)}^{2}\right)}^{0.2}=0.7200\text{.}$$

$$\prod_{i=1}^{n}{\left(1+\left(\lambda -1\right)\left(1-\theta_{I_{i}}^{q}\right)\right)}^{w_{i}}={\left(1+\left(2-1\right){(1-0.4}^{2})\right)}^{0.3}{\left(1+\left(2-1\right)\left({1-0.3}^{2}\right)\right)}^{0.1}{\left(1+\left(2-1\right){(1-0.7}^{2})\right)}^{0.4}{(1+\left(2-1\right){(1-0.6}^{2}))}^{0.2}=1.6136\text{.}$$

$$\left(\lambda -1\right)\prod_{i=1}^{n}{\left(\vartheta_{I_{i}}^{q}\right)}^{w_{i}}=\left(2-1\right)\left(0.4^{0.3}0.3^{0.1}0.7^{0.4}0.6^{0.2}\right)=0.1784\text{.}$$

$$=\left(\frac{\sqrt[{p}]{\frac{\prod_{i=1}^{n}{\left(1+\left(\lambda -1\right)\vartheta_{I_{i}}^{p}\right)}^{w_{i}}-\prod_{i=1}^{n}{\left(1-\vartheta_{I_{i}}^{p}\right)}^{w_{i}}}{\prod_{i=1}^{n}{\left(1+\left(1-\lambda \right)\vartheta_{I_{i}}^{p}\right)}^{w_{i}}+\left(\lambda -1\right)\prod_{i=1}^{n}{\left(1-\vartheta_{I_{i}}^{p}\right)}^{w_{i}}},\sqrt[{q}]{\lambda }\prod_{i=1}^{n}\vartheta_{I_{i}}^{w_{i}}}}{\sqrt[{q}]{\prod_{i=1}^{n}{\left(1+\left(\lambda -1\right)\left(1-\theta_{I_{i}}^{q}\right)\right)}^{w_{i}}+}\left(\lambda -1\right)\prod_{i=1}^{n}{\left(\theta_{I_{i}}^{q}\right)}^{w_{i}}}\right)=\left(\sqrt[{2}]{\frac{1.2929-0.7200}{1.2929+0.7200},\frac{\left(1.4142\right)\left(0.1784\right)}{\sqrt[{2}]{1.6136-.1784}}}\right)\text{.}$$

=(0.5335,0.2105).

### 3.3 Properties of p,q –QOFHWA operator

The proposed operator p,q –QOFHWA possesses several significant properties. Outlined as follows:

Property 1 When a collection of p,q –QOFNs is denoted as $I_{i}=\left(\vartheta_{I_{i}},\theta_{I_{i}}\right)$ (i=1,2,…,n), and if they are all equivalent, meaning $I_{i}=\left(\vartheta_{I_{i}},\theta_{I_{i}}\right)=I_{i}=\left(\vartheta_{I},\theta_{I}\right)$ for all i, then it follows that p,q –QOFHWA $\left(I_{1},I_{2},\ldots ,I_{n}\right)=I=\left(\vartheta_{I},\theta_{I}\right)$.

**Proof.** Given that $I_{i}=\left(\vartheta_{I_{i}},\theta_{I_{i}}\right)$ for i=1,2,…,n, employing Equation (9) leads to the following:

$$\left(\begin{array}{c} \sqrt[{p}]{\frac{\prod_{i=1}^{n}{\left(1+\left(\lambda -1\right)\vartheta_{I_{i}}^{p}\right)}^{w_{i}}-\prod_{i=1}^{n}{\left(1-\vartheta_{I_{i}}^{p}\right)}^{w_{i}}}{\prod_{i=1}^{n}{\left(1+\left(1-\lambda \right)\vartheta_{I_{i}}^{p}\right)}^{w_{i}}+\left(\lambda -1\right)\prod_{i=1}^{n}{\left(1-\vartheta_{I_{i}}^{p}\right)}^{w_{i}}},}\\ \frac{\sqrt[{q}]{\lambda }\prod_{i=1}^{n}\vartheta_{I_{i}}^{w_{i}}}{\sqrt[{q}]{\prod_{i=1}^{n}{\left(1+\left(\lambda -1\right)\left(1-\theta_{I_{i}}^{q}\right)\right)}^{w_{i}}+}\left(\lambda -1\right)\prod_{i=1}^{n}{\left(\theta_{I_{i}}^{q}\right)}^{w_{i}}} \end{array}\right)$$

Since $\left(I_{1},I_{2},\ldots ,I_{n}\right)=I=\left(\vartheta_{I},\theta_{I}\right)$ then, we get

$$\left(\begin{array}{c} \sqrt[{p}]{\frac{{\left(1+\left(\lambda -1\right)\vartheta_{I_{i}}^{p}\right)}^{w_{i}}-{\left(1-\vartheta_{I_{i}}^{p}\right)}^{w_{i}}}{{\left(1+\left(1-\lambda \right)\vartheta_{I_{i}}^{p}\right)}^{w_{i}}+\left(\lambda -1\right){\left(1-\vartheta_{I_{i}}^{p}\right)}^{w_{i}}},}\\ \;\frac{\sqrt[{q}]{\lambda }\vartheta_{I_{i}}^{w_{i}}}{\sqrt[{q}]{{(1+\left(\lambda -1\right)(1-\theta_{I_{i}}^{q}))}^{w_{i}}+}\left(\lambda -1\right){\left(\theta_{I_{i}}^{q}\right)}^{w_{i}}} \end{array}\right)=I=\left(\vartheta_{I},\theta_{I}\right)\text{.}$$

Property 2 Let $I_{i}=\left(\vartheta_{I_{i}},\theta_{I_{i}}\right)$ for i=1,2,…,n be a collection of p,q−QOFNs. Let $I^{-}=\min\left(I_{i}\right)$ and $I^{+}=\max\left(I_{i}\right)$. Then $I^{-}≼p,q-\text{QOFHWA}\left(I_{1},I_{2},\ldots I_{n}\right)≼I^{+}\text{.}$Where $\min\;I_{i}=\left(\min\left(\vartheta_{I_{i}}\right),\max\left(\theta_{I_{i}}\right)\right)$, $\max\;I_{i}=\left(\max\left(\vartheta_{I_{i}}\right),\min\left(\theta_{I_{i}}\right)\right)$ for all i=1,2,…,n.

Let $I_{1}=\left(0.5,0.3\right)$ and $I_{2}=\left(0.4,0.2\right)$ are two p,q− QOFNs where p=q=3, λ=2 and $w_{1}=w_{2}=0.5$, then $I^{-}=\left(0.4,0.3\right)$, $I^{+}=\left(0.5,0.2\right)$ and $p,q-\text{QOFHWA}\left(I_{1},I_{2}\right)=\left(0.3439,0.2718\right)$. By using score function we get

$\text{Sc}\left(I^{-}\right)=\frac{1+\vartheta_{I^{-}}^{p}-\theta_{I^{+}}^{p}}{2}=\frac{1+0.40^{3}-0.30^{3}}{2}=0.5185$, $\text{Sc}\left(I^{-}\right)=\frac{1+\vartheta_{I^{-}}^{p}-\theta_{I^{+}}^{p}}{2}=\frac{1+0.50^{3}-0.20^{3}}{2}=0.5585$ and

$Sc\left(\;p,q-QOFHWA\left(I_{1},I_{2}\right)\right)=\frac{1+{0=0.4239}^{3}-0.39018^{3}}{2}=0.5243$. Therefore, by Definition 7, we have $I^{-}≼p,q-QOFHWA\left(I_{1},I_{2},\ldots I_{n}\right)≼I^{+}$.

Property 3 Assume that $I_{i}=\left(\vartheta_{I_{i}}\right)$ for i=1,2,…,n and $I_{i}^{*}=\left(\vartheta_{I_{i}}^{*},\theta_{I_{i}}^{*}\right)$ are two families of p,q− QOFNs such that $I_{i}≼I_{i}^{*}\text{,}$ then p,q− QOFHWA $\left(I_{1},I_{2},\ldots I_{n}\right)≼p,q-$ QOFHWA $\left(I_{1}^{*},I_{2}^{*},\ldots ,I_{n}^{*}\right)\text{.}$

### 3.4 Special cases

When λ=1, it follows that p,q− QOFHWA is equivalent to p,q− QOF weighted averaging (p,q− QOFWA) [16] and Equation (10) showcases the mathematical formulation.

(10) $$p,q-\;\text{QOFWA}\;\left(I_{1},I_{2},\ldots I_{n},\right)=\left(\sqrt[{p}]{1-\Pi_{i=1}^{n}{\left(1-\vartheta_{I_{i}}^{p}\right)}^{w_{i}}}\right),\Pi_{i-1}^{n}{\left({\theta_{I}}_{i}\right)}^{w_{i}}$$

When λ=1, p,q− QOFHWA transforms into the p,q− QOF Einstein weighted averaging (p,q− QPFEWA) operator and can be represented as follows:

(11) $$\left(\sqrt[{p}]{\frac{\Pi_{i=1}^{n}{\left(1+\vartheta_{I_{i}}^{p}\right)}^{w_{i}}-\Pi_{i=1}^{n}{\left(1-\vartheta_{I_{i}}^{p}\right)}^{w_{i}}}{\Pi_{i=1}^{n}{\left(1+\vartheta_{I_{i}}^{p}\right)}^{w_{i}}+\left(\lambda -1\right)\Pi_{i=1}^{n}{\left(1-\vartheta_{I_{i}}^{p}\right)}^{w_{i}}},}\frac{\sqrt[{q}]{2}\Pi_{i=1}^{n}\theta_{I_{i}}^{w_{i}}}{\sqrt[{q}]{\Pi_{i=1}^{n}{\left(1+\left(1-\theta_{I_{i}}^{q}\right)\right)}^{w_{i}}+\Pi_{i=1}^{n}{\left(\theta_{I_{i}}^{q}\right)}^{w_{i}}}}\right)$$

### **3.5** p,q−**QOFHWG operators**

Definition 11 Considering a collection of p,q− QOFNs denoted as $I_{i}=\left(\vartheta_{I_{i}},\theta_{I_{i}}\right)$ (i=1,2,…,n),

Then $p,q-QOFHWA:I^{n}\to I$ and can be defined as follows:

(12) $$p,q-QOFHWA\left(I_{1},I_{2},\ldots ,I_{n}\right){⨂}_{i=1}^{n}\left(w_{i}I_{i}\right)=\left(\begin{array}{c} \frac{\sqrt[{p}]{\lambda }\Pi_{i=1}^{n}\vartheta_{I_{i}}^{w_{i}}}{\sqrt[{p}]{\Pi_{i=1}^{n}{\left(1+\left(\lambda -1\right)\left(1-\vartheta_{I_{i}}^{p}\right)\right)}^{w_{i}}+\left(\lambda -1\right)\Pi_{i=1}^{n}{\left(\vartheta_{I_{i}}^{p}\right)}^{w_{i}}}},\\ \sqrt[{q}]{\frac{\Pi_{i=1}^{n}{\left(1+\left(\lambda -1\right)\left(\theta_{I_{i}}^{p}\right)\right)}^{w_{i}}-\Pi_{i=1}^{n}{\left(1-\theta_{I_{i}}^{q}\right)}^{w_{i}}}{\Pi_{i=1}^{n}{\left(1+\left(1-\lambda \right)\theta_{I_{i}}^{q}\right)}^{w_{i}}+\left(\lambda -1\right)\Pi_{i=1}^{n}{\left(1-\theta_{I_{i}}^{q}\right)}^{w_{i}}}} \end{array}\right)$$

Where $w={\left(w_{1},w_{2},\ldots ,w_{n}\right)}^{T}$ indicates the weight vector of $I_{i}=\left(\vartheta_{I_{i}},\theta_{I_{i}}\right)\;\left(i=1,2,\ldots ,n\right)$ following the conditions that $w_{i}≻0$ and the constraints $\Sigma_{i=1}^{n}w_{i}=1$.

Theorem 2 For any collection of p,q− QOFNs represented as $I_{i}=\left(\vartheta_{I_{i}},\theta_{I_{i}}\right)\left(i=1,2,\ldots ,n\right)$, the aggregated value obtained by p,q− QOFHWG operator, are also a p,q− QOFNs.

***Proof*.** The demonstration of this proof can be easily accomplished by employing mathematical induction based on the variable n.

***Step* 1.** When n=1, the value of $w_{1}$ become 1. And upon evaluating the lift side of Equation (12), we get

p,q− QOFHWG $\left(I_{1},I_{2},\ldots ,I_{n}\right)=I_{1}=\left(\vartheta_{I_{i}},\theta_{I_{i}}\right)$, also, for the right-hand side of Equation (12), we obtain:

$$\left(\frac{\sqrt[{p}]{\lambda }\vartheta_{I_{1}}}{\sqrt[{p}]{1+\left(\lambda -1\right)\left(1-\vartheta_{I_{1}}^{p}\right)+\left(\lambda -1\right)\vartheta_{I_{1}}^{p}}},\sqrt[{q}]{\frac{1+\left(\lambda -1\right)\vartheta_{I_{1}}^{q}-\left(1-\vartheta_{I_{1}}^{q}\right)}{1+\left(1-\lambda \right)\vartheta_{I_{1}}^{q}+\left(\lambda -1\right)\left(1-\vartheta_{I_{1}}^{q}\right)}}\right)=\left(\vartheta_{I_{i}},\theta_{I_{i}}\right)\text{.}$$

Thus, Equation (12) is valid when n=1.

***Step* 2.** Assume that Equation (12) is valid for the situation where n=k, where k is any real number, given this assumption, Equation (11) can be represented as:

$$p,q-QOFHWG\left(I_{1},I_{2},\ldots ,I_{k}\right){⨂}_{i=1}^{k}(w_{i}I_{i}=\left(\begin{array}{c} \frac{\sqrt[{p}]{\lambda }\Pi_{i=1}^{k}\vartheta_{I_{i}}^{w_{i}}}{\sqrt[{p}]{\Pi_{i=1}^{k}{\left(1+\left(\lambda -1\right)\left(1-\vartheta_{I_{1}}^{p}\right)\right)}^{w_{i}}+\left(\lambda -1\right)\Pi_{i=1}^{n}{\left(\vartheta_{I_{i}}^{p}\right)}^{w_{i}}}},\\ \sqrt[{q}]{\frac{\Pi_{i=1}^{k}{\left(1+\left(\lambda -1\right)\left(\theta_{I_{1}}^{q}\right)\right)}^{w_{i}}-\Pi_{i=1}^{k}{\left(1-\theta_{I_{i}}^{q}\right)}^{w_{i}}}{\Pi_{i=1}^{k}{\left(1+\left(1-\lambda \right)\theta_{I_{i}}^{q}\right)}^{w_{i}}+\left(\lambda -1\right)\Pi_{i=1}^{k}{\left(1-\theta_{I_{i}}^{q}\right)}^{w_{i}}}} \end{array}\right)$$

***Step* 3.** Now, when k=n+1, we examine the following equation.

$$p,q-QOFHWG\left(I_{1},I_{2},\ldots I_{k}\right)={⨂}_{i=1}^{k}\left(w_{i}I_{i}\right)⨂\;\left(w_{k+1}I_{k+1}\;\right)$$

$$=\left(\begin{array}{c} \frac{\sqrt[{p}]{\lambda }\Pi_{i=1}^{k}\vartheta_{I_{i}}^{w_{i}}}{\sqrt[{p}]{\Pi_{i=1}^{k}{\left(1+\left(\lambda -1\right)\left(1-\vartheta_{I_{i}}^{p}\right)\right)}^{w_{i}}+\left(\lambda -1\right)\Pi_{i=1}^{n}{\left(\vartheta_{I_{i}}^{p}\right)}^{w_{i}}}},\\ \sqrt[{q}]{\frac{\Pi_{i=1}^{k}{\left(1+\left(\lambda -1\right)\left(\theta_{I_{i}}^{q}\right)\right)}^{w_{i}}-\Pi_{i=1}^{k}{\left(1-\theta_{I_{i}}^{q}\right)}^{w_{i}}}{\Pi_{i=1}^{k}{\left(1+\left(1-\lambda \right)\theta_{I_{i}}^{q}\right)}^{w_{i}}+\left(\lambda -1\right)\Pi_{i=1}^{k}{\left(1-\theta_{I_{i}}^{q}\right)}^{w_{i}}}} \end{array}\right)⊗\left(\begin{array}{c} \frac{\sqrt[{p}]{\lambda }\vartheta_{I_{i+1}}^{w_{i+1}}}{\sqrt[{p}]{{\left(1+\left(\lambda -1\right)\left(1-\vartheta_{I_{k+1}}^{p}\right)\right)}^{w_{k+1}}+\left(\lambda -1\right){\left(\vartheta_{I_{k+1}}^{p}\right)}^{w_{k+1}}}},\\ \sqrt[{q}]{\frac{{\left(1+\left(\lambda -1\right)\left(\theta_{I_{k+1}}^{q}\right)\right)}^{w_{k+1}}-{\left(1-\theta_{I_{k+1}}^{q}\right)}^{w_{k+1}}}{{\left(1+\left(1-\lambda \right)\theta_{I_{k+1}}^{q}\right)}^{w_{k+1}}+\left(\lambda -1\right){\left(1-\theta_{I_{k+1}}^{q}\right)}^{w_{k+1}}}} \end{array}\right)$$

$$=\left(\begin{array}{c} \frac{\sqrt[{p}]{\lambda }\Pi_{i=1}^{k}\vartheta_{I_{i}}^{w_{i}}}{\sqrt[{p}]{\Pi_{i=1}^{k+1}{\left(1+\left(\lambda -1\right)\left(1-\vartheta_{I_{i}}^{p}\right)\right)}^{w_{i}}+\left(\lambda -1\right)\Pi_{i=1}^{k+1}{\left(\vartheta_{I_{i}}^{p}\right)}^{w_{i}}}},\\ \sqrt[{q}]{\frac{\Pi_{i=1}^{k+1}{\left(1+\left(\lambda -1\right)\left(\theta_{I_{i}}^{q}\right)\right)}^{w_{i}}-\Pi_{i=1}^{k+1}{\left(1-\theta_{I_{i}}^{q}\right)}^{w_{i}}}{\Pi_{i=1}^{k+1}{\left(1+\left(1-\lambda \right)\theta_{I_{i}}^{q}\right)}^{w_{i}}+\left(\lambda -1\right)\Pi_{i=1}^{k+1}{\left(1-\theta_{I_{i}}^{q}\right)}^{w_{i}}}} \end{array}\right)\text{.}$$

*As a result*, *it can be inferred that* equation (12) *holds for the case of* k=n+1. *By combining the insights from steps* (1), (2), *and* (3), *it becomes evident that this outcome extends to any values of* n *within the set of natural numbers*.

Example 4 For any four p,q –QOFNs represented as $I_{1}=\left(0.3,0.2\right).\;I_{2}=\left(0.4,0.3\right),I_{3}=\left(0.4,0.2\right)\;and\;I_{4}=\left(0.6,0.5\right)\text{.}$ Let $w={\left(0.1,0.3,0.2,0.4\right)}^{T}$ represent the weight vector for these p,q –QOFNs. We assume λ=3 and p=q=4. Then

$$\sqrt[{p}]{\lambda }\prod_{i=1}^{n}\vartheta_{I_{i}}^{w_{i}}=\sqrt[{4}]{3}\left(0.3^{0.1}\times 0.4^{0.3}\times 0.4^{0.2}\times 0.6^{0.4}\right)=0.6016$$

$$\prod_{i=1}^{n}{(1+\left(\lambda -1\right)(1-\vartheta_{I_{i}}^{p}))}^{w_{i}}={\left(1+\left(3-1\right){\left(1-0.3\right)}^{4}\right)}^{0.1}{\left(1+\left(3-1\right){\left(1-0.4\right)}^{4}\right)}^{0.3}{\left(1+\left(3\;1\right){\left(1-0.4\right)}^{4}\right)}^{0.2}{\left(1+\left(3-1\right){\left(1-0.6\right)}^{4}\right)}^{0.4}=1.1261\text{,}$$

${\left(1-\vartheta_{I_{i}}^{p}\right)}^{w_{i}}={\left(1-{\left(0.2\right)}^{4}\right)}^{0.1}{\left(1-{\left(0.3\right)}^{4}\right)}^{0.3}{\left(1-{\left(0.2\right)}^{4}\right)}^{0.2}{\left(1-{\left(0.5\right)}^{4}\right)}^{0.4}=0.9142$, and hence

$$\left(\begin{array}{c} \frac{\sqrt[{p}]{\lambda }\prod_{i=1}^{n}\vartheta_{I_{i}}^{w_{i}}}{\sqrt[{p}]{\prod_{i=1}^{n}{\left(1+\left(\lambda -1\right)\left(1-\vartheta_{I_{i}}^{p}\right)\right)}^{w_{i}}+\left(\lambda -1\right)\prod_{i=1}^{n}{\left(\vartheta_{I_{i}}^{p}\right)}^{w_{i}}}},\\ \sqrt[{q}]{\frac{\prod_{i=1}^{n}{\left(1+\left(\lambda -1\right)\left(\theta_{I_{i}}^{q}\right)\right)}^{w_{i}}+\left(\lambda -1\right)\prod_{i=1}^{n}{\left(1-\theta_{I_{i}}^{q}\right)}^{w_{i}}}{\prod_{i=1}^{n}{\left(1+\left(1-\lambda \right)\theta_{I_{i}}^{q}\right)}^{w_{i}}+\left(\lambda -1\right)\prod_{i=1}^{n}{\left(1-\theta_{I_{i}}^{q}\right)}^{w_{i}}}} \end{array}\right)=\left(\frac{0.6016}{\sqrt[{4}]{1.1906-0.9142}},\sqrt[{4}]{\frac{1.2011-0.7234}{1.2011+0.7234}}\right)=\left(0.8296,0.\;7251\right)\text{.}$$

### 3.6 Properties of p,q –QOFHWG operator

The proposed operator p,q –QOFHWG holds numerous significant properties, outlined as follows:

Property 4 For any collection of p,q –QOFNs denoted as $I_{i}=\left(\vartheta_{I_{i}},\theta_{I_{i}}\right)$ for i=1,2,…,n, and if they are all equal, meaning $I_{i}=\left(\vartheta_{I_{i}},\theta_{I_{i}}\right)=\left(\vartheta_{I},\theta_{I}\right)$ for all i, then it follows that p,q –QOFHWG $\left(I_{1},I_{2},\ldots ,I_{n}\right)=I=\left(\vartheta_{I},\theta_{I}\right)\text{.}$

***Proof***. For any collection $I_{i}=\left(\vartheta_{I_{i}},\theta_{I_{i}}\right)\;\text{for}\;i=1,2,\ldots ,n$ utilizing Equation (11) leads to the following:

$$\left(\frac{\sqrt[{p}]{\lambda }\prod_{i=1}^{n}\vartheta_{I_{i}}^{w_{i}}}{\sqrt[{p}]{\prod_{i=1}^{n}{\left(1+\left(\lambda -1\right)\left(1-\vartheta_{I_{i}}^{p}\right)\right)}^{w_{i}}+\left(\lambda -1\right)\prod_{i=1}^{n}{\left(\vartheta_{I_{i}}^{p}\right)}^{w_{i}}}},\sqrt[{q}]{\frac{\prod_{i=1}^{n}{\left(1+\left(\lambda -1\right)\left(\theta_{I_{i}}^{q}\right)\right)}^{w_{i}}+\prod_{i=1}^{n}{\left(1-\theta_{I_{i}}^{q}\right)}^{w_{i}}}{\prod_{i=1}^{n}{\left(1+\left(1-\lambda \right)\theta_{I_{i}}^{q}\right)}^{w_{i}}+\left(\lambda -1\right)\prod_{i=1}^{n}{\left(1-\theta_{I_{i}}^{q}\right)}^{w_{i}}}}\right)$$

$$=\left(\frac{\sqrt[{p}]{\lambda }\;\vartheta_{I}}{\sqrt[{p}]{\left(1+\left(\lambda -1\right)\left(1-\vartheta_{I}^{p}\right)\right)+\left(\lambda -1\right)\vartheta_{I}^{p}}},\sqrt[{q}]{\frac{1+\left(\lambda -1\right)\theta_{I}^{q}+\left(1-\theta_{I}^{q}\right)}{1+\left(1-\lambda \right)\theta_{I}^{q}+\left(\lambda -1\right)1-\theta_{I}^{q}}}\;\right)=I=\left(\vartheta_{I},\theta_{I}\right)\text{.}$$

Property 5 Let $I_{i}=\left(\vartheta_{I_{i}},\theta_{I_{i}}\right)\;\text{for}\;i=1,2,\ldots ,n$ be a collection of p,q –QOFNs. Let $I^{-}=\min\left(I_{i}\right)I^{+}=\max\;\left(I_{i}\right)$. It can be established that $I^{-}≼p,q$ – QOFHWG $\left(I_{1},I_{2},\ldots ,I_{n}\right)≼I^{+}$

Property 6 Let $I_{i}=\left(\vartheta_{I_{i}},\theta_{I_{i}}\right)\;\text{for}\;i=1,2,\ldots ,n$ and $I_{i}^{*}=\left(\vartheta_{i}^{*},\theta_{i}^{*}\right)$ are two families of p,q –QOFNs such that $I_{i}≼I_{i}^{*}$, then it can be deduced that p,q – QOFHWG $\left(I_{1},I_{2},\ldots ,I_{n}\right)≼p,q$ – QOFHWG $\left(I_{1}^{*},I_{2}^{*},\ldots ,I_{n}^{*}\right)$.

### 3.7 Special cases of p,q – QOFHWG operator

When λ=1, it follows that p,q – QOFHWG is equivalent to p,q – QOF weighted averaging (p,q–QOFHWG) operator [19]. The mathematical formulation of p,q–QOFHWG operator is presented in Equation (13).

(13) $$p,q–\text{QOFHWG}\;\left(I_{1},I_{2},\ldots ,I_{n}\right)=\left(\prod_{i=1}^{n}{\left(\theta_{I_{i}}\right)}^{w_{i}}\right),\sqrt[{q}]{1-\prod_{i=1}^{n}{\left(1-\theta_{I_{i}}^{q}\right)}^{w_{i}}}$$

When λ=1, p,q – QOFHWG operator transforms into the p,q – QOF Einstein weighted geometric (p,q – QOFHWG) operator and presented in Equation (14):

(14) $$\left(\begin{array}{c} \frac{\sqrt[{p}]{\lambda }\prod_{i=1}^{n}\vartheta_{I_{i}}^{w_{i}}}{\sqrt[{p}]{\prod_{i=1}^{n}{\left(1+\left(1-\vartheta_{I_{i}}^{p}\right)\right)}^{w_{i}}+\prod_{i=1}^{n}{\left(\vartheta_{I_{i}}^{p}\right)}^{w_{i}}}},\\ \sqrt[{q}]{\frac{\prod_{i=1}^{n}{(1+\theta_{I_{i}}^{q}))}^{w_{i}}+\prod_{i=1}^{n}{\left(1-\theta_{I_{i}}^{q}\right)}^{w_{i}}}{\prod_{i=1}^{n}{\left(1+\theta_{I_{i}}^{q}\right)}^{w_{i}}+\left(\lambda -1\right)\prod_{i=1}^{n}{\left(1-\theta_{I_{i}}^{q}\right)}^{w_{i}}}} \end{array}\right)$$

## 4 MCDM approach based on suggested AOs

This section of the paper introduces a novel approach to MCDM that leverages p,q – QOFHWA and p,q – QOFHWG operators to effectively handle scenarios where the information provided by the decision makers are in the form of p,q – QOFNs. Let $Z=\left\{Z_{1},Z_{2},\ldots ,Z_{m}\right\}$ denotes the collection of m alternatives, and $C=\left\{C_{1},C_{2},\ldots ,C_{n}\right\}$ represent the set of n criteria associated with each alternative. Here $Z_{i}$ signifies the $i^{th}$ alternative, and $C_{j}$ represent $j^{th}$ criterian. Moreover, let $D=\left\{D_{1},D_{2},\ldots ,D_{h}\right\}$ be a set comprising t experts, all of whom provide their inputs in the form of p,q – QOFNs. The weight vector for experts is $\rho =\left\{\rho_{1},\rho_{2},\ldots ,\rho_{h}\right\}$, adhering to the condition that $\rho_{\xi }\in \left[0,1\right]$ for all ξ=1,2,…,h and $\sum_{\xi =1}^{h}\rho_{\xi }=1\text{.}$ Similarly, the weight vector corresponding to each criterion is represented as $w={\left(w_{1},w_{2},\ldots ,w_{n}\right)}^{T}$ where $w_{j}\in \left[0,1\right]$ and $\sum_{j=1}^{n}w_{j}=1\text{.}$ The information contributed by the experts relative to each criterion is depicted through p,q – QOF decision matrices, represented as $\varepsilon^{h}={\left(e_{ij}^{h}\right)}_{m\times n}$.

### 4.1 Algorithm

**Step 1.** Gather information represented by p,q – QOFNs about the alternative associated with criteria in the decision matrix as follows:

(15) $$\varepsilon^{h}=\left(\begin{array}{c} \left(\vartheta_{I_{11}},\theta_{I_{11}}\right)\\ \left(\vartheta_{I_{21}},\theta_{I_{21}}\right)\\ \vdots \\ \left(\vartheta_{I_{m1}},\theta_{I_{m1}}\right) \end{array}\begin{array}{c} \left(\vartheta_{I_{12}},\theta_{I_{12}}\right)\ldots \\ \left(\vartheta_{I_{22}},\theta_{I_{22}}\right)\ldots \\ \vdots \\ \left(\vartheta_{I_{m2}},\theta_{I_{m2}}\right)\ldots \end{array}\begin{array}{c} \left(\vartheta_{I_{1n}},\theta_{I_{1n}}\right)\\ \left(\vartheta_{I_{2n}},\theta_{I_{2n}}\right)\\ \vdots \\ \left(\vartheta_{I_{mn}},\theta_{I_{mn}}\right) \end{array}\right)$$

**Step 2.** In the decision-making process, criteria play a crucial role in evaluating and comparing alternatives to make informed choices. Two common types of criteria used in decision-making are cost criteria and benefit criteria. Cost criteria involve that represent expenditures, expenses, or negative outcomes associated with the alternative under consideration. These criteria are used to assess the financial implications and potential drawbacks of each alternative. Benefits criteria. On the other hand, encompass positive outcomes, gains, or advantages associated with the alternatives. These criteria help assess the potential benefits, advantages, or positive impact of each alternative. When the decision matrix (listed in Equation (15)) incorporates both type of criteria, it becomes necessary to normalize the matrix using the following formula presented in Equation (16).

(16) $${\tilde{e}}_{ij}^{h}=\left\{ \begin{array}{c} \left(\vartheta_{I_{ij}},\theta_{I_{ij}}\right)\;\text{Benefit}\;\text{type}\;\\ \left(\theta_{I_{ij}},\vartheta_{I_{ij}}\right)\;\text{Cost}\;\text{Type}\; \end{array}\right.$$

**Step 3.** In MSDM problems, the importance of weights assigned to criteria and decision makers cannot be overstated. These weights play a crucial role in achieving a balanced assessment of criteria, enabling effective comparison of alternatives, and ensuring objective decision making. By quantifying the relative significance of criteria and incorporating the preferences of decision makers, the weight assignment process in MCDM contributes to a structured, informed, and well-rounded decision-making approach that aligns with the objectives and dynamics of the decision problems at hand. Utilizing the entropy measure of p,q–QOFSs. We introduce an innovative approach for determining criteria weights with formulation given in Equation (17):

(17) $$w_{i}=\frac{1-B_{\left(e_{j}\right)}}{n-\sum_{j=1}^{n}B_{\left(e_{j}\right)}}$$

Where $B_{\left(e_{j}\right)}=1-d\left(e_{j},{e_{j}}^{c}\right)$.

Example 4. Let $e_{1}=\left(0.3,0.4\right)\text{,}$ and $e_{2}=\left(0.5,0.2\right)$, then ${e_{1}}^{c}=\left(0.4,0.3\right)\text{,}$ and ${e_{2}}^{c}=\left(0.2,0.5\right)$. We assume p=q=2, then:

$$B\left(e_{1}\right)=1-\left(\left|0.3^{2}-0.4^{2}\right|+\left|0.4^{2}-0.3^{2}\right|\right)=1-\left(\left|0.09-0.16\right|+\left|0.16-0.09\right|\right)=1-\left(\left|0.07-0.07\right|\right)=0.86$$

$$B\left(e_{2}\right)=1-\left(\left|0.5^{2}-0.2^{2}\right|+\left|0.2^{2}-0.5^{2}\right|\right)=1-\left(\left|0.25-0.04\right|+\left|0.04-0.25\right|\right)=1-\left(\left|0.21-0.21\right|\right)=0.58$$

Now, from Equation (16), we have

$$w_{1}=\frac{1-0.86}{2-1.44}=0.25,\text{and}\;w_{2}=\frac{1-0.58}{2-1.44}=0.75\text{.}$$

**Fig. 2:**
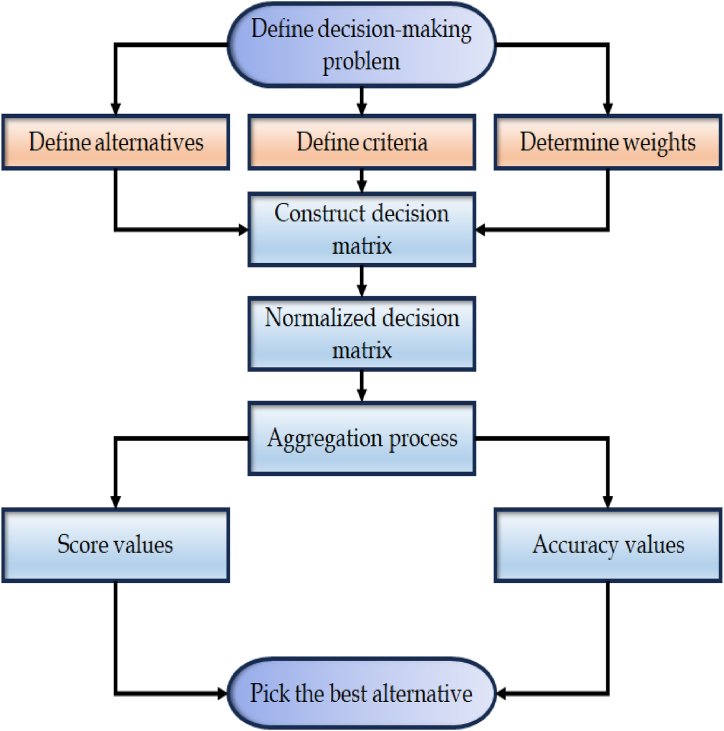
Flowchart for the proposed model.

**Step 4.** Aggregate the rating values of each alternative through the application of proposed AOs.
**Step 5.** Calculate the score values of each alternative by using Equation (4).
**Step 6.** Rank the alternatives according to the score values. The flowchart of the proposed model is presented in Fig. 2.

## 5 Case study

**Fig. 3:**
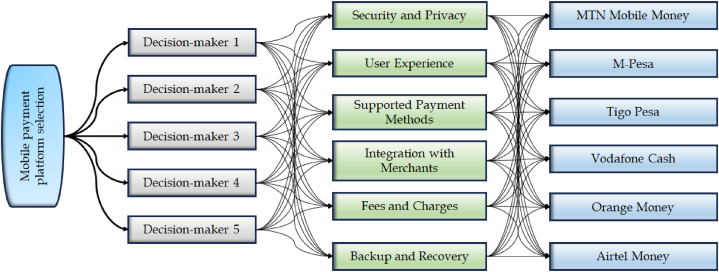
Tree diagram depicting the proposed model.

The progression of disruptive technologies has ushered in new era for mobile devices, equipping them with novel functionalities that cater to multitude of mobile financial services encompassing bill payments, account transfers, person-to-person proximity payments, exchanges, remote payments, and a spectrum of other services like mobile ticketing and discounts [37]. In this landscape, the emergence of “mobile payment technology” stands out as a swiftly evolving phenomenon within the diverse array of contemporary mobile applications. Mobile payment can be defined as an interface bridging mobile devices and payment mechanisms, facilitating transactions conducted through these devices. Notably, in sub- Saharan Africa, the adoption of mobile payment technology has left an impressive imprint, underscored by various operators providing a wide range of services in this dynamic field. With the positive influence that mobile payment impacts on organizations, a multitude of mobile payment Platforms have emerged on a global scale. Evaluating and choosing among these platforms can be intricate due to the need for meticulous identification. Moreover, given the uncertainties and vagueness prevalent in real-world scenarios, decision-makers often face challenges in accurately scrutinizing mobile payment platforms. However, in the resolution to this proposed decision-making approach, a practical instance involving the selection of a specific mobile payment platform is employed as an illustrative example. A group of five decision-makers (experts) $\left\{D_{1},D_{2},D_{3},D_{4},D_{5}\right\}$, has been convened to assess six distinct mobile payment platforms (alternatives), namely MTN Mobile Money ($Z_{1}$), M-Pesa $\left(Z_{2}\right)$ Tigo Pesa $\left(Z_{3}\right)\text{,}$ Vodafone Cash $\left(Z_{4}\right)$, Orange Money $\left(Z_{5}\right)$, and Airtel Money $\left(Z_{6}\right)$, We consider six important criteria: Security and Privacy $\left(C_{1}\right)$, User Experience $\left(C_{2}\right)\text{,}$ Supported Payment Methods $\left(C_{3}\right)$, Integration with Merchants $\left(C_{4}\right)\text{,}$ Fees and Charges $\left(C_{5}\right)\text{,}$ and Backup and Recovery $\left(C_{6}\right)$. The weight vector for the factors is established using Equation (12), yielding w=(0.2132,0.1876,0.1524,0.1038,0.2287,1143). The assessment scores of alternatives based on the criteria specified by the decision-makers are outlined in Table 1, Table 2, Table 3, Table 4, Table 5. Fig. 3 illustrates the tree diagram depicting the structure of the proposed model.

**Table 1:** Information provided by $D_{1}$.

$Z_{i}$	$C_{1}$	$C_{2}$	$C_{3}$	$C_{4}$	$C_{5}$	$C_{6}$
$Z_{1}$	(0.30,0.40)	(0.20,0.50)	(0.40,0.50)	(0.35,0.30)	(0.45,0.40)	(0.55,0.30)
$Z_{2}$	(0.20,0.25)	(0.15,0.30)	(0.40,0.20)	(0.30,0.35)	(0.25,0.20)	(0.10,0.40)
$Z_{3}$	(0.30,0.10)	(0.50,0.30)	(0.60,0.40)	(0.55,0.30)	(0.30,0.15)	(0.50,0.20)
$Z_{4}$	(0.20,0.25)	(0.50,0.70)	(0.20,0.15)	(0.30,0.75)	(0.25,0.45)	(0.30,0.35)
$Z_{5}$	(0.30,0.40)	(0.25,0.50)	(0.40,0.20)	(0.45,0.30)	(0.35,0.55)	(0.45,0.50)
$Z_{6}$	(0.50,0.60)	(0.35,0.30)	(0.45,0.50)	(0.15,0.60)	(0.60,0.20)	(0.50,0.30)

**Table 2:** Information provided by $D_{2}$.

$Z_{i}$	$C_{1}$	$C_{2}$	$C_{3}$	$C_{4}$	$C_{5}$	$C_{6}$
$Z_{1}$	(0.30,0.15)	(0.20,0.60)	(0.50,0.15)	(0.70,0.60)	(0.30,0.10)	(0.20,0.75)
$Z_{2}$	(0.60,0.50)	(0.20,0.40)	(0.60,0.50)	(0.50,0.45)	(0.30,0.60)	(0.50,0.70)
$Z_{3}$	(0.30,0.10)	(0.60,0.20)	(0.75,0.35)	(0.65,0.40)	(0.45,0.20)	(0.55,0.10)
$Z_{4}$	(0.35,0.55)	(0.30,0.60)	(0.40,0.75)	(0.45,0.20)	(0.45,0.60)	(0.55,0.40)
$Z_{5}$	(0.45,0.10)	(0.70,0.75)	(0.30,0.40)	(0.30,0.15)	(0.30,0.15)	(0.45,0.40)
$Z_{6}$	(0.40,0.60)	(0.35,0.70)	(0.35,0.40)	(0.20,0.90)	(0.90,0.60)	(0.30,0.5)

**Table 3:** Information provided by $D_{3}$.

$Z_{i}$	$C_{1}$	$C_{2}$	$C_{3}$	$C_{4}$	$C_{5}$	$C_{6}$
$Z_{1}$	(0.60,0.70)	(0.50,0.60)	(0.55,0.30)	(0.20,0.40)	(0.50,0.60)	(0.40,0.30)
$Z_{2}$	(0.45,0.30)	(0.55,0.60)	(0.70,0.75)	(0.40,0.50)	(0.55,0.70)	(0.50,0.45)
$Z_{3}$	(0.30,0.20)	(0.40,0.30)	(0.65,0.10)	(0.75,0.60)	(0.75,0.65)	(0.80,0.40)
$Z_{4}$	(0.50,0.60)	(0.20,0.60)	(0.70,0.60)	(0.85,0.30)	(0.70,0.60)	(0.20,0.60)
$Z_{5}$	(0.70,0.30)	(0.40,0.55)	(0.45,0.35)	(0.45,0.60)	(0.40,0.65)	(0.70,0.55)
$Z_{6}$	(0.30,0.40)	(0.55,0.70)	(0.80,0.60)	(0.40,0.90)	(0.50,0.75)	(0.30,0.10)

**Table 4:** Information provided by $D_{4}$.

$Z_{i}$	$C_{1}$	$C_{2}$	$C_{3}$	$C_{4}$	$C_{5}$	$C_{6}$
$Z_{1}$	(0.30,0.10)	(0.20,0.60)	(0.55,0.30)	(0.40,0.30)	(0.50,0.55)	(0.80,0.50)
$Z_{2}$	(0.50,0.60)	(0.50,0.75)	(0.60,0.70)	(0.50,0.60)	(0.30,0.40)	(0.40,0.30)
$Z_{3}$	(0.75,0.30)	(0.50,0.15)	(0.40,0.10)	(0.40,0.30)	(0.90,0.30)	(0.50,0.30)
$Z_{4}$	(0.45,0.50)	(0.30,0.35)	(0.40,0.45)	(0.35,0.45)	(0.30,0.50)	(0.40,0.55)
$Z_{5}$	(0.60,0.70)	(0.50,0.65)	(0.60,0.50)	(0.50,0.40)	(0.70,0.60)	(0.70,0.30)
$Z_{6}$	(0.30,0.35)	(0.45,0.60)	(0.55,0.45)	(0.60,0.30)	(0.45,0.60)	(0.20,0.60)

**Table 5:** Information provided by $D_{5}$.

$Z_{i}$	$C_{1}$	$C_{2}$	$C_{3}$	$C_{4}$	$C_{5}$	$C_{6}$
$Z_{1}$	(0.35,0.50)	(0.30,0.45)	(0.50,0.40)	(0.30,0.10)	(0.50,0.40)	(0.40,0.20)
$Z_{2}$	(0.30,0.10)	(0.30,0.20)	(0.50,0.60)	(0.30,0.50)	(0.30,0.70)	(0.50,0.40)
$Z_{3}$	(0.40,0.30)	(0.55,0.10)	(0.60,0.35)	(0.70,0.55)	(0.75,0.30)	(0.40,0.60)
$Z_{4}$	(0.35,0.60)	(0.65,0.35)	(0.30,0.45)	(0.45,0.60)	(0.30,0.50)	(0.55,0.60)
$Z_{5}$	(0.40,0.60)	(0.20,0.45)	(0.55,0.70)	(0.30,0.50)	(0.35,0.60)	(0.45,0.40)
$Z_{6}$	(0.10,0.40)	(0.30,0.55)	(0.40,0.35)	(0.60,0.65)	(0.80,0.70)	(0.20,0.30)

We take the weight vector of decision-makers to be (0.25,0.20,0.15,0.30,0.10). Aggregate the data provided by the decision-makers through the utilization of p,q− QOFHWA operator, with the specific settings of p=q=4 and a Hamacher parameter value of λ=2. A summary of aggregated values can be found in Table 6.

**Table 6:** Aggregated values.

$Z_{i}$	$C_{1}$	$C_{2}$	$C_{3}$
$Z_{1}$	(0.3917,0.4341)	(0.4492,0.4832)	(0.3809,0.4518)
$Z_{2}$	(0.3165,0.3346)	(0.4723,0.4315)	(0.5193,0.4684)
$Z_{3}$	(0.5262,0.2716)	(0.4589,0.3166)	(0.5372,0.3754)
$Z_{4}$	(0.2519,0.3874)	(0.2867,0.3291)	(0.4176,0.3371)
$Z_{5}$	(0.3549,0.4604)	(0.3351,0.4126)	(0.4305,0.2968)
$Z_{6}$	(0.4255,0.4883)	(0.2993,0.3458)	(0.3014,0.4695)
	$C_{4}$	$C_{5}$	$C_{6}$
$Z_{1}$	(0.3422,0.4156)	(0.4103,0.5146)	(0.4270,0.3911)
$Z_{2}$	(0.4119,0.3916)	(0.4285,0.2814)	(0.4677,0.2983)
$Z_{3}$	(0.4597,0.3181)	(0.4873,0.2736)	(0.5123,0.3486)
$Z_{4}$	(0.2984,0.4253)	(0.3457,0.3987)	(0.3616,0.4142)
$Z_{5}$	(0.4321,0.3552)	(0.3872,0.2987)	(0.4522,0.3524)
$Z_{6}$	(0.4256,0.2315)	(0.2844,9.3137)	(0.4489,0.3287)

We utilized p,q− QOFHWA operator to consolidate the previously aggregated information presented in Table 6, with the parameter values p=q=4, λ=2 and criteria weights are w=(0.2132,0.1876,0.1524,0.1038,0.2287,0.1143) as follows:

$$\prod_{i=1}^{n}{\left(1-\left(\lambda -1\right)\vartheta_{I_{i}}^{p}\right)}^{w_{i}}=\left(1+\left(2-1\right){\left(0.3917^{4}\right)}^{0.2132}\right)\left(1+\left(2-1\right){\left(0.4492\right)}^{0.1876}\right)1+\left(2-1\right){\left(0.3909^{4}\right)}^{0.1524}$$

$$1+\left(2-1\right){\left(0.3422^{4}\right)}^{0.1083}1+\left(2-1\right){\left(0.4103^{4}\right)}^{0.2287}1+\left(2-1\right){\left(0.4270^{4}\right)}^{0.1143}=1.0275\text{.}$$

$$\prod_{i=1}^{n}{\left(1-\vartheta_{I_{i}}^{p}\right)}^{w_{i}}={\left(1-0.3919^{4}\right)}^{0.2132}{\left(1-0.4492^{4}\right)}^{0.1876}{\left(1-0.3809^{4}\right)}^{0.1524}{\left(1-0.3422^{4}\right)}^{0.1083}$$

$${\left(1-0.4103^{4}\right)}^{0.2287}{\left(1-0.3911^{4}\right)}^{0.1143}=0.9724\text{.}$$

$$\Pi_{i=1}^{I}\theta_{I_{i}}^{w_{i}}={\left(0.4341\right)}^{0.2132}\times {\left(0.4832\right)}^{0.1876}\times {\left(0.4518\right)}^{0.1524}\times {\left(0.4156\right)}^{1083}\times {\left(0.5146\right)}^{0.2287}\times {\left(0.3911\right)}^{0.1143}=0.4557\text{.}$$

$$\Pi_{i=1}^{n}{\left(1+\left(\lambda -1\right)\left(1-\theta_{I_{1}}^{q}\right)\right)}^{w_{i}}={\left(1+\left(2-1\right){\left(1-0.4341\right)}^{4}\right)}^{0.2132}\times {\left(1+\left(2-1\right){\left(1-0.4832\right)}^{4}\right)}^{0.1876}$$

$$\times {\left(1+\left(2-1\right){\left(1-0.4518\right)}^{4}\right)}^{0.1524}\times {\left(1+\left(2-1\right){\left(1-0.4156\right)}^{4}\right)}^{0.1083}\times {\left(1+\left(2-1\right){\left(1-0.5146\right)}^{4}\right)}^{0.2287}$$

$$\times {\left(1+\left(2-1\right){\left(1-0.3911\right)}^{4}\right)}^{0.1143}=1.9540\text{.}$$

$$=\left(\begin{array}{c} \sqrt[{p}]{\frac{\Pi_{i=1}^{n}{\left(1+\left(\lambda -1\right)\left(\vartheta_{I_{i}}^{p}\right)\right)}^{w_{i}}-\Pi_{i=1}^{n}{\left(1-\vartheta_{I_{i}}^{p}\right)}^{w_{i}}}{\Pi_{i=1}^{n}{\left(1+\left(1-\lambda \right)\theta_{I_{i}}^{p}\right)}^{w_{i}}+\left(\lambda -1\right)\Pi_{i=1}^{n}{\left(1-\theta_{I_{i}}^{p}\right)}^{w_{i}}}},\\ \frac{\sqrt[{q}]{\lambda }\Pi_{i=1}^{n}\theta_{I_{i}}^{w_{i}}}{\sqrt[{q}]{\Pi_{i=1}^{n}{\left(1+\left(\lambda -1\right)\left(1-\theta_{I_{i}}^{p}\right)\right)}^{w_{i}}+\left(\lambda -1\right)\Pi_{i=1}^{n}{\left(\theta_{I_{i}}^{p}\right)}^{w_{i}}}} \end{array}\right)=\left(\sqrt[{4}]{\frac{1.0275-0.9724}{1.0275+\left(2-1\right)\left(0.9724\right)}},\frac{\sqrt[{4}]{2}\left(0.4557\right)}{\sqrt[{4}]{1.9540+\left(2-1\right)\left(0.4557\right)}}\right)=\left(4074,4350\right)\text{.}$$

The aggregated values are summarized in Table 7.

**Table 7:** **Aggregated values for**p=q=4**, and**λ=4.

Alternatives	Aggregated value
$Z_{1}$	(0.4074,0.4350)
$Z_{2}$	(0.4436,0.3427)
$Z_{3}$	(0.5006,0.2971)
$Z_{4}$	(0.3364,0.3616)
$Z_{5}$	(0.3823,0.3469)
$Z_{6}$	(0.3727,0.3498)

We can determine the score values of the alternatives by applying Equation (4) as follows:

$$Sc\left(Z_{1}\right)=\frac{1+\vartheta_{I}^{p}-\theta_{I}^{q}}{2}=Sc\left(Z_{1}\right)=\frac{1+0.4074^{4}-0.4350^{4}}{2}=0.4959\text{.}$$

$$Sc\left(Z_{2}\right)=\frac{1+\vartheta_{I}^{p}-\theta_{I}^{q}}{2}=Sc\left(Z_{2}\right)=\frac{1+0.4436\;-0.3427^{4}}{2}=0.5018\text{.}$$

$$Sc\left(Z_{3}\right)=\frac{1+\vartheta_{I}^{p}-\theta_{I}^{q}}{2}=Sc\left(Z_{3}\right)=\frac{1+0.5006^{4}-0.2971^{4}}{2}=0.5275\text{.}$$

$$Sc\left(Z_{4}\right)=\frac{1+\vartheta_{I}^{p}-\theta_{I}^{q}}{2}=Sc\left(Z_{4}\right)=\frac{1+0.3364^{4}-0.3616^{4}}{2}=0.4979\text{.}$$

$$Sc\left(Z_{5}\right)=\frac{1+\vartheta_{I}^{p}-\theta_{I}^{q}}{2}=Sc\left(Z_{5}\right)=\frac{1+0.3823^{4}-0.3469^{4}}{2}=0.5034\text{.}$$

$$Sc\left(Z_{6}\right)=\frac{1+\vartheta_{I}^{p}-\theta_{I}^{q}}{2}=Sc\left(Z_{6}\right)=\frac{1+0.4074^{4}-0.3498^{4}}{2}=0.5022\text{.}$$

Based on the score values, the ranking order of the alternatives is as follows: $Z_{3}≻Z_{5}≻Z_{6}≻Z_{2}≻Z_{4}≻Z_{1}$**.** According to the rating values, $Z_{3}$**,** which corresponds to Tigo Pesa, emerges as the most optimal choice.

Now, we employ p,q− QOFHWG operator to consolidate the rating values of alternatives. The score values and the resulting ranking order of alternatives through p,q− QOFHWG operator (p=q=3;λ=3) are presented in Table 8.

**Table 8:** Ranking order of alternatives for p=q=3, and λ=3.

**Operator**	p	q	λ	**Score values**					
				$Z_{1}$	$Z_{2}$	$Z_{3}$	$Z_{4}$	$Z_{5}$	$Z_{6}$
p,q−**QOFHWG**	3	3	3	0.4197	0.4409	0.4538	0.4231	0.4451	0.4417

Observing Tables 8 and it's evident that the order of alternatives in term of ranking remain consistent with the ranking acquired through the p,q− QOFHWA operator. Consequently, p,q− QOFHWG operator can be considered as a viable alternative aggregation operator for these assessments.

### 5.1 Sensitivity analysis

To illustrate the influence of the parameter λ on the decision outcomes, we maintain fixed values for p and q at 4 while varying λ from 1 to 15. The resulting score values and the corresponding ranking order of alternatives have been tabulated in Table 9. From Tables 9 and it becomes evident that the optimal choice of alternative remains consistent across different values of λ. However, it is noteworthy that alternatives $Z_{3}$ and $Z_{2}$ share the same ranking position, while the positions of the remaining alternatives exhibit variations as λ ranges from 1 to 5. Subsequently, the ranking order stabilizes from scenario 6 to 15, suggesting a convergence of preferences among the decision-makers. This analysis underscores the impact of parameter λ on the ranking dynamics of the alternatives, particularly during the initial range of values (1 to 5), after which the rankings become consistent and stable. On the other hand, it has been observed that as the parameter value increases, the score values of alternatives also increase, implying an optimistic perspective for decision-makers.

**Table 9:** Score values and ranking order of alternatives for different values of parameter λ.

λ	p	q	**Score values**						**Best Alternative**
			$Z_{1}$	$Z_{2}$	$Z_{3}$	$Z_{4}$	$Z_{5}$	$Z_{6}$	$Z_{3}$
1	4	4	0.4774	0.4981	0.5094	0.4886	0.4938	0.4921	$Z_{3}$
2	4	4	0.4959	0.5125	0.5275	0.4979	0.5050	0.5022	$Z_{3}$
3	4	4	0.5075	0.5235	0.5429	0.5035	0.5130	0.5090	$Z_{3}$
4	4	4	0.5168	0.5332	0.5568	0.5079	0.5199	0.5147	$Z_{3}$
5	4	4	0.5248	0.5422	0.5698	0.5118	0.5262	0.5198	$Z_{3}$
6	4	4	0.5321	0.5505	0.5819	0.5153	0.5322	0.5246	$Z_{3}$
7	4	4	0.5389	0.5584	0.5932	0.5187	0.5378	0.5291	$Z_{3}$
8	4	4	0.5454	0.5660	0.6040	0.5218	0.5432	0.5335	$Z_{3}$
9	4	4	0.5515	0.5732	0.6141	0.5249	0.5484	0.5377	$Z_{3}$
10	4	4	0.5574	0.5801	0.6237	0.5279	0.5535	0.5417	$Z_{3}$
11	4	4	0.5630	0.5868	0.6329	0.5308	0.5584	0.5457	$Z_{3}$
12	4	4	0.5685	0.5932	0.6415	0.5336	0.5632	0.5495	$Z_{3}$
13	4	4	0.5738	0.5993	0.6498	0.5364	0.5679	0.5532	$Z_{3}$
14	4	4	0.5790	0.6053	0.6577	0.5391	0.5724	0.5568	$Z_{3}$
15	4	4	0.5840	0.6110	0.6653	0.5418	0.5769	0.5604	$Z_{3}$

**Fig. 4:**
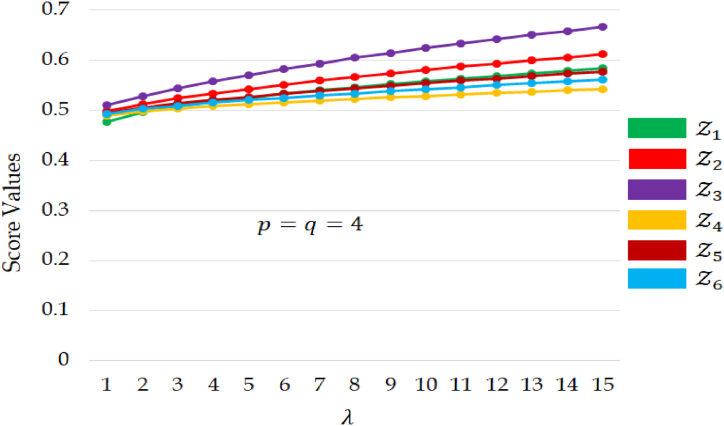
The variation of alternatives for different values of λ.

Consequently, when decision-makers harbor optimism, they may assign lower values to the parameter, resulting in a decrease in the score values of the overall alternatives. Importantly, it's noteworthy that the best alternative remains unchanged, emphasizing the objectivity and stability of the results, impervious to the preferences of decision-makers for either pessimism or optimism. The impact of the parameter λ over raking order is visually depicted in Fig. 4.

**Fig. 5:**
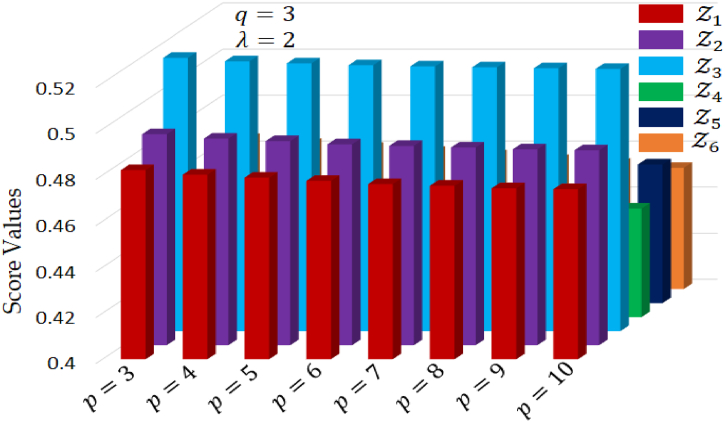
Ranking of alternatives for different values of p, while fixing q=3, and λ=2.

In this section, we aim to illustrate how varying parameters p and q can influence the outcomes of our decision process. In this scenario, we have kept the Hamacher parameter λ fixed at 2. Table 10 provides a comprehensive display of score values and the resulting ranking order of alternatives under different values of p, with q set at a constant value of 3. Analyzing Tables 10 and it becomes evident that while the score values differ across various p values, the overall ranking order remains consistent throughout these variations. Fig. 5 illustrates a graphical representation of alternatives under varying parameter p, while keeping q fixed at 3 and λ at a constant value of 2.

**Table 10:** Score values and ranking order for different values of p where q=3 and λ=2.

p	q	**Score values**						**Ranking order**
		$Z_{1}$	$Z_{2}$	$Z_{3}$	$Z_{4}$	$Z_{5}$	$Z_{6}$	
**3**	3	0.4822	0.4917	0.5188	0.4576	0.4697	0.4652	$Z_{3}≻Z_{2}≻Z_{1}≻Z_{5}≻Z_{6}≻Z_{4}$
**4**	3	0.4802	0.4898	0.5173	0.4564	0.4676	0.4630	$Z_{3}≻Z_{2}≻Z_{1}≻Z_{5}≻Z_{6}≻Z_{4}$
**5**	3	0.4789	0.4887	0.5164	0.4552	0.4664	0.4613	$Z_{3}≻Z_{2}≻Z_{1}≻Z_{5}≻Z_{6}≻Z_{4}$
**6**	3	0.4775	0.4873	0.5156	0.4529	0.4655	0.4596	$Z_{3}≻Z_{2}≻Z_{1}≻Z_{5}≻Z_{6}≻Z_{4}$
**7**	3	0.4761	0.4866	0.5151	0.4511	0.4641	0.4581	$Z_{3}≻Z_{2}≻Z_{1}≻Z_{5}≻Z_{6}≻Z_{4}$
**8**	3	0.4754	0.4859	0.5147	0.4497	0.4628	0.4560	$Z_{3}≻Z_{2}≻Z_{1}≻Z_{5}≻Z_{6}≻Z_{4}$
**9**	3	0.4743	0.4851	0.5143	0.4482	0.4617	0.4544	$Z_{3}≻Z_{2}≻Z_{1}≻Z_{5}≻Z_{6}≻Z_{4}$
**10**	3	0.4739	0.4847	0.5140	0.4471	0.4602	0.4526	$Z_{3}≻Z_{2}≻Z_{1}≻Z_{5}≻Z_{6}≻Z_{4}$

From the earlier conversation, it becomes evident that by adjusting the parameter p we gain the facility to manage and modulate the influence of membership status of an alternative in the evaluation process. This parameter enables us to fine-tune and manage the way an alternative's membership affects the overall evaluation.

Likewise, Table 11 displays the score values and ranking order of alternatives for different values of q, with p set at a constant value of 3 and λ maintained at 2. Examining this table shows that, even though the score values vary for various q values, the relative ranking order of the alternatives remains consistent during these variations.

**Table 11:** Score values and ranking order for different values of q where p=3 and λ=2.

p	q	**Score values**						**Ranking order**
		$Z_{1}$	$Z_{2}$	$Z_{3}$	$Z_{4}$	$Z_{5}$	$Z_{6}$	
3	3	0.4822	0.4917	0.5188	0.4576	0.4697	0.4652	$Z_{3}≻Z_{2}≻Z_{1}≻Z_{5}≻Z_{6}≻Z_{4}$
4	3	0.4809	0.4908	0.5182	0.4568	0.4688	0.4636	$Z_{3}≻Z_{2}≻Z_{1}≻Z_{5}≻Z_{6}≻Z_{4}$
5	3	0.4801	0.4900	0.5176	0.4563	0.4679	0.4628	$Z_{3}≻Z_{2}≻Z_{1}≻Z_{5}≻Z_{6}≻Z_{4}$
6	3	0.4796	0.4791	0.5169	0.4551	0.4673	0.4623	$Z_{3}≻Z_{2}≻Z_{1}≻Z_{5}≻Z_{6}≻Z_{4}$
7	3	0.4793	0.4783	0.5162	0.4546	0.4667	0.4617	$Z_{3}≻Z_{2}≻Z_{1}≻Z_{5}≻Z_{6}≻Z_{4}$
8	3	0.4790	0.4777	0.5158	0.4539	0.4660	0.4615	$Z_{3}≻Z_{2}≻Z_{1}≻Z_{5}≻Z_{6}≻Z_{4}$
9	3	0.4788	0.4769	0.5155	0.4533	0.4654	0.4609	$Z_{3}≻Z_{2}≻Z_{1}≻Z_{5}≻Z_{6}≻Z_{4}$
10	3	0.4786	0.4763	0.5152	0.4529	0.4647	0.4606	$Z_{3}≻Z_{2}≻Z_{1}≻Z_{5}≻Z_{6}≻Z_{4}$

**Fig. 6:**
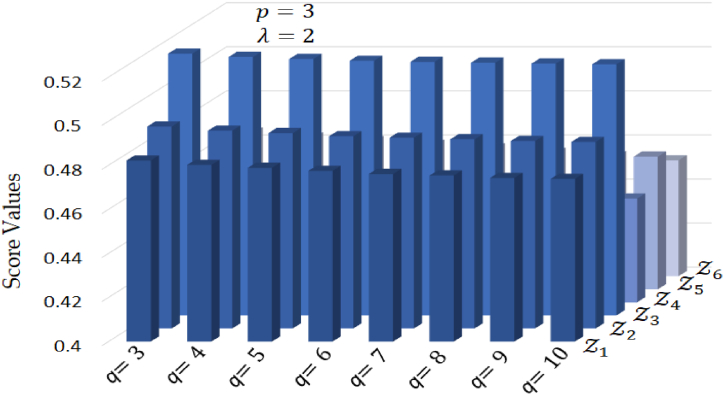
Ranking order of alternatives for different values of q.

From this discussion, it became clear that when we adjust parameter q, we acquire the capability to effectively regulate and fine-tune the influence of an alternative non-membership status within the assessment process. This parameter acts as a significant tool that enables us to finely manage how an alternative's non-membership status participates in the overall evaluation, allowing for a more nuanced and tailored approach to decision-making. The graphical view of alternatives for various values of parameter q is displayed in Fig. 6.

### 5.3 Comparative analysis

In this section, we undertake a comprehensive comparison analysis between the proposed approach and several existing approaches. Through this analysis, we aim to establish the validity and effectiveness of our approach by highlighting its performance of established methods. The data provided by the decision-makers in Table 1, Table 2, Table 3, Table 4, Table 5 cannot be effectively assessed using aggregation operators within the Intuitionistic fuzzy or Pythagorean fuzzy framework. These limitations arise because 0.80+0.70≻1 and $0.80^{2}+0.70^{2}≻1$. Such situations fall outside the scope of these fuzzy environments, as they violate the fundamental principles and constraints of Intuitionistic fuzzy and Pythagorean fuzzy sets. Hence, we explore alternative methods grounded in Fermatean fuzzy sets [26,36,[38], [39], [40], [41]] or q− rung orthopair fuzzy sets [14,15] to serve as benchmarks for comparing with our proposed approach. The score values with these approaches are listed in Table 12.

**Table 12:** Comparison with existing approaches.

Approaches	Score values						Best alternative
	$Z_{1\;}$	$Z_{2}$	$Z_{3\;}$	$Z_{4\;}$	$Z_{5\;}$	$Z_{6\;}$	
Hadi et al. [26]	0.3953	0.4372	0.4511	0.4068	0.4184	0.4217	$Z_{3\;}$
Garg et al. [38]	0.5181	0.5375	0.5686	0.5294	0.5209	0.5332	$Z_{3\;}$
Rani and Mishra [39]	0.3246	0.3494	0.3516	0.3423	0.3390	0.3471	$Z_{3\;}$
Chakraborty and Saha [40]	0.4164	0.4212	0.4419	0.4337	0.4214	0.4383	$Z_{3\;}$
Liu and Wang [36]	0.5627	0.5855	0.6024	0.5777	0.5863	0.5310	$Z_{3\;}$
Peng et al. [15]	0.2709	0.2980	0.3167	0.2945	0.2698	0.2775	$Z_{3\;}$
Jana et al. [14]	0.6483	0.6825	0.7382	0.7025	0.7119	0.6384	$Z_{3\;}$
Wei et al. [41]	0.4369	0.4611	0.4897	0.4728	0.4547	0.4486	$Z_{3\;}$

**Fig. 7:**
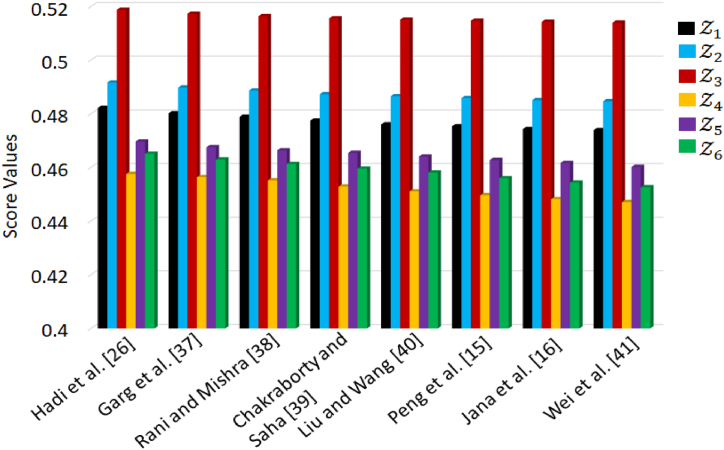
Score values for alternatives with different approaches.

From the data presented in Tables 12 and it's apparent that the ranking order of the alternatives exhibits slight variations. However, it's noteworthy that the outcome remains consistent, as Alternative $Z_{3}$ maintains its position as the top-rated choice across these different rankings. In contrast to the conventional methods in use, the proposed decision-making approach with the p,q− QOF framework offers a significantly enhanced level of flexibility to decision-makers. It empowers them to finely control the evaluation of alternatives by considering the parameters p and q. In contrast, existing methods cannot adjust membership and non-membership degrees within the decision-making process, as they operate without such parameters. Fig. 7 displays a graphical representation of the score values for alternatives using different approaches. Fig. 7. Score values for alternatives with different approaches.

### 5.4 Advantages

The proposed approach offers several advantages:

1. The introduction of parameters p and q in the aggregation operators provides decision-makers with a high degree of flexibility. They can adjust these parameters according to the specific requirements and preferences of the decision-making scenarios. This adaptability of the decision process can account for various degrees of membership and non-membership, making it applicable to a wide range of real-world situations.
2. The parametric nature of the proposed operators allows decision-makers to fine-tune the influence of membership and non-membership degrees. This level of control empowers decision-makers to precisely tailor the aggregation process to their preferences and the specifics of the problem at hand.
3. The symmetry of the proposed aggregation operators concerning the parameter y ensures that the ranking orders of alternatives remain relatively stable across different parameter values. This objective is crucial in decision-making, as it prevents results from being swayed by decisionmakers' pessimism or optimism.

### 5.5 Limitations

While the proposed approach offers several advantages, it's important to acknowledge its limitations:

4. Although the parametric nature of the approach provides flexibility, it also introduces the need to choose appropriate values (p, q, λ). The sensitivity of resto these parameters can be a drawback, as selecting incorrect values may lead to biased or less reliable outcomes.
5. The introduction of multiple parameters increases the complexity of the decision-making process.

## 6 Conclusion and future work

This paper presents a novel framework for MCDM using a series of aggregation operators constructed within the p,q− quasirung orthopair fuzzy environments. These operators are formulated based on Hamacher t-norm and t-conorm, offering a parametric approach that allows fine-tuning the aggregation process to accommodate decision-makers’ preferences. The research explores the fundamental properties of these operators and introduces a practical MCDM approach utilizing p,q− quasirung orthopair fuzzy numbers. A real-world scenario involving the selection of mobile payment platforms is used to illustrate the proposed method, and its results are compared with some existing approaches to validate its effectiveness. The study conducts sensitivity analysis and employs graphical representations to showcase the resilience and practicality of the proposed approach in various contexts. Additionally, the paper discusses the advantages and limitations of the proposed framework, shedding light on its potential and areas for further development.

## Funding

This research received no funding.

## Informed consent statement

Not applicable.

## Data availability

The accompanying manuscript does not contain any associated data. The paper only presents the written text and does not have any additional data that supports the claims and conclusions presented in the manuscript.

## CRediT authorship contribution statement

**Touqeer Ahmad:** Validation, Methodology, Investigation, Conceptualization. **Muhammad Rahim:** Writing – review & editing, Writing – original draft, Formal analysis, Conceptualization. **Jie Yang:** Validation, Software, Formal analysis, Data curation. **Rabab Alharbi:** Methodology, Formal analysis, Data curation. **Hamiden Abd El-Wahed Khalifa:** Validation, Software, Formal analysis, Data curation.

## Declaration of competing interest

We, the authors of the paper titled “Development of p,q− quasirung orthopair Fuzzy Hamacher Aggregation operators and its application in Decision-making Problems**”**, hereby declare that we have no financial or non-financial interests that could be perceived as influencing the objectivity or integrity of the work presented. We affirm that we have not received any external funding that may have biased the outcomes or conclusions of the paper. We confirm that the ideas, concepts, and intellectual property presented in the paper are original and do not infringe upon the rights of any third party. We acknowledge our responsibility to promptly disclose any actual or perceived conflicts of interest that may arise during the publication process. We understand that providing this declaration ensures transparency and upholds the credibility of our work.
